# A Generalized Kinetic Model for Coupling between Stepping and ATP Hydrolysis of Kinesin Molecular Motors

**DOI:** 10.3390/ijms20194911

**Published:** 2019-10-03

**Authors:** Ping Xie, Si-Kao Guo, Hong Chen

**Affiliations:** 1 School of Materials Science and Energy Engineering, FoShan University, Guangdong 528000, China; hchen2017@163.com; 2 Key Laboratory of Soft Matter Physics, Institute of Physics, Chinese Academy of Sciences, Beijing 100190, China; th_ink@163.com

**Keywords:** molecular motor, kinesin, mechanochemistry, ATPase activity, dynamics, model

## Abstract

A general kinetic model is presented for the chemomechanical coupling of dimeric kinesin molecular motors with and without extension of their neck linkers (NLs). A peculiar feature of the model is that the rate constants of ATPase activity of a kinesin head are independent of the strain on its NL, implying that the heads of the wild-type kinesin dimer and the mutant with extension of its NLs have the same force-independent rate constants of the ATPase activity. Based on the model, an analytical theory is presented on the force dependence of the dynamics of kinesin dimers with and without extension of their NLs at saturating ATP. With only a few adjustable parameters, diverse available single molecule data on the dynamics of various kinesin dimers, such as wild-type kinesin-1, kinesin-1 with mutated residues in the NLs, kinesin-1 with extension of the NLs and wild-type kinesin-2, under varying force and ATP concentration, can be reproduced very well. Additionally, we compare the power production among different kinesin dimers, showing that the mutation in the NLs reduces the power production and the extension of the NLs further reduces the power production.

## 1. Introduction

Motor proteins, also called molecular motors, are important classes of macromolecules that play critical roles in supporting and maintaining various biological processes [1,2]. Kinesins are a superfamily of motor proteins capable of moving on microtubule (MT) filaments by using the energy from ATP hydrolysis to perform diverse functions such as the transport of intracellular cargoes, responsibility of chromosome segregation during cell division, etc. [3,4,5,6,7,8,9]. Among the kinesin superfamily [10], kinesin-1 and kinesin-2 motors are two typical families that have been studied extensively and intensively. A kinesin-1 motor has two identical N-terminal heads that are connected together by a coiled-coil stalk through their neck linkers (NLs) (each consisting of 14 amino acid residues) [11]. An example of kinesin-2 motors is vertebrate KIF17 that is also a homodimer but with the NL in each head having 17 residues [12]. Both families of kinesin dimers can move stepwise and progressively on MT filaments in a hand-over-hand manner and toward the plus end of MT in about 8.2 nm increments—the periodicity of an MT filament [13]. Important and interesting issues related to the motors are how the chemical reaction of ATPase activity couples with the mechanical stepping (i.e., the chemomechanical coupling) and their movement dynamics [14].

With various experimental methods, the dynamics of the kinesin motors has been investigated in detail. In particular, using single-molecule optical trappings the dependences of the velocity upon the external force acting on the coiled-coil stalk and upon the ATP concentration were determined [15,16,17,18,19,20,21,22,23,24,25,26]. The velocity decreases with the increase of the external force resisting the forward movement (called backward force). The velocity increases with the increase of ATP concentration, satisfying the Michaelis–Menten kinetics. Furthermore, it was revealed that extending the NLs of kinesin-1 dimer such as human kinesin-1 reduces both the velocity and stall force [26]. More puzzlingly, the experimental data revealed that under no external force, multiple ATP molecules are hydrolyzed per mechanical step for the human kinesin-1 dimer with extension of the NLs [26] while for the wild-type (WT) case about one ATP molecule is consumed per mechanical step [15,27]. To explain these experimental data, a lot of models have been proposed in the literature [14,26,28,29,30,31,32,33,34,35,36]. Most of the models argued that the ATPase activity of the kinesin motor is regulated by the strain on the NLs. In those models, it was proposed that the external backward force, which affects the strain on the NLs, results in the reduction of the ATPase rate and thus the velocity, and the extension of the NLs, which also affects the strain on the NLs, reduces the ATPase rate and thus the velocity. Recently, with a simplified model proposing that the external force does not affect the rate constants of ATPase activity we studied the dynamics of WT kinesin-1 dimers under varying external force, reproducing quantitatively diverse available single-molecule data for WT kinesin-1 dimers [37,38]. Using complicated numerical simulations of the mechanical motion coupled with ATPase activity, the dynamics of the WT dimers and that of the mutant ones with extension of their NLs were investigated [39,40]. Nevertheless, an analytical and simple theory is desirable to explain consistently both the dynamics of the WT dimers and that of the mutants with extension of their NLs.

In this work, inspired by previous studies [37,38,39,40], we present a general kinetic model for the chemomechanical coupling pathway to study the dynamics of both WT and mutant kinesin dimers with extension of their NLs under varying external force and ATP concentration. In our model, the rate constants of ATPase activity are independent of both the external force on the NLs and the length of the NLs. Based on the model, we study analytically the dynamics of kinesin dimers with and without extension of their NLs at saturating ATP and study numerically the dynamics at non-saturating ATP. With only a few adjustable parameters, the theoretical and numerical data explain quantitatively the diverse available single-molecule data on kinesin-1 dimers with and without extension of their NLs as well as kinesin-2 KIF17. Moreover, to compare performance among different species of kinesin dimers we study analytically their power production and efficiency, showing that the power production and efficiency by the mutant dimer are greatly reduced compared to the WT dimer.

## 2. Results and Discussion

### 2.1. The Model

We first describe the model for the chemomechanical coupling pathway of kinesin dimer, based on which our theoretical analyses are made. The model is built up on the basis of the following three pieces of experimental and computational evidence and/or arguments.

(1) A kinesin head in nucleotide-free (ϕ), ATP, or ADP.Pi state binds strongly to MT whereas in ADP state binds weakly to MT, as experimental data showed [41,42,43,44,45]. Additionally, immediately after Pi release there is a very short time period *t*_r_ (in the order of μs) when the ADP-head has a lower affinity to the local binding site (denoted by *E*_w1_) on MT than that to other binding sites (denoted by *E*_w2_), as all-atom molecular dynamics (MD) simulations indicated [46]. This can be understood as follows [46,47]. The large conformational changes in the local MT tubulin induced by the strong interaction with ϕ-, ATP- or ADP.Pi-head [48] result in the local tubulin having a further weaker interaction with the ADP-head than other unperturbed tubulins. As the weak interaction between the ADP-head and MT can hardly induce the conformational change of the MT-tubulin [46], in the time *t*_r_ after Pi release from the MT-bound ADP.Pi head the local MT-tubulin returns to the normally unchanged conformation, with the binding energy of the local MT-tubulin to the ADP-head becoming the same as other tubulins.

(2) When an MT-bound head is in ATP or ADP.Pi state, its NL can dock into its motor domain whereas in ϕ or ADP state its NL cannot dock [49,50]. Structural analyses showed that the NL docking involves NL strand β9 forming a cover-neck bundle with strand β0 of the motor domain [22,51], which can occur when the two strands are in proximity.

(3) An interaction exists between the MT-bound head and detached ADP-head. When the MT-bound head is in ADP or ϕ state, with undocked NL, the two heads have a high binding energy, while when the MT-bound head is in ATP or ADP.Pi state, with docked NL, the binding energy is reduced greatly, as recent all-atom MD simulations indicated (see Figure S1 in Supplementary Information, which is reproduced from Shi et al., to be published in Proteins).

**Figure 1:**
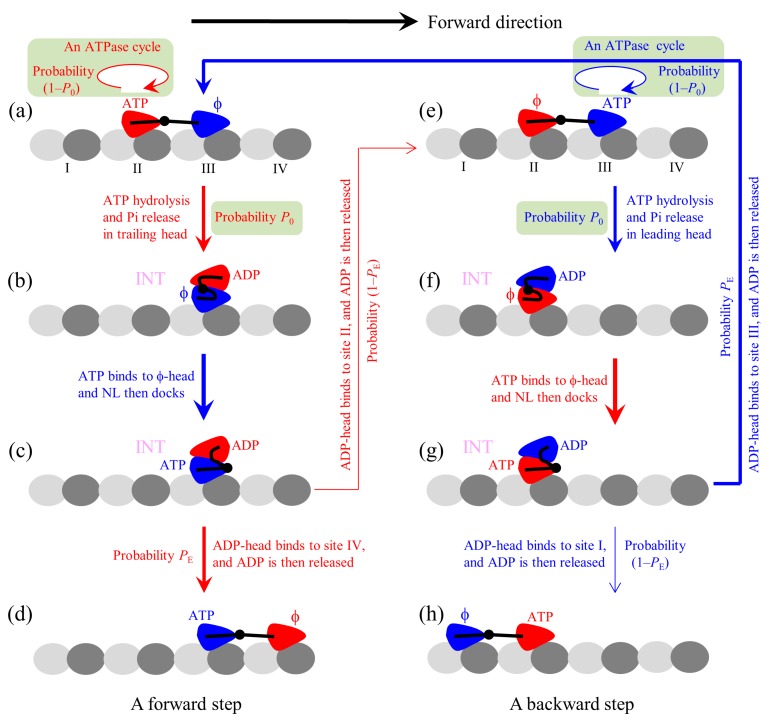
The model of stepping of kinesin dimer at low ATP concentrations. (**a**–**h**) The pathway for the chemomechanical coupling (see text for detailed description). The thickness of the arrow represents the magnitude of the transition probability under no external force.

The proposed chemomechanical coupling pathway at low ATP is schematically illustrated in Figure 1. We begin with the trailing head in ATP state binding strongly to binding site II on MT while the leading head in ϕ state binding strongly to site III (Figure 1a). After an ATPase activity (ATP hydrolysis and Pi release) taking place in the trailing head, the ADP-head has a low affinity (*E*_w1_) to the local site II for a very short time period *t*_r_ in the order of μs. During *t*_r_, it is most probable (with a probability *P*_0_) that the ADP-head escapes from the potential well of depth *E*_w1_ and then diffuses to the intermediate (INT) position relative to the MT-bound head, where the two heads have a high binding energy (Figure 1b). If the ADP-head has not escaped from the potential well of depth *E*_w1_ within *t*_r_ (with probability 1–*P*_0_), after the depth of the potential well changes to *E*_w2_ the ADP-head is almost unable to escape from the potential well until ADP is released. Then, ATP binding, ATP hydrolysis, and Pi release take place in the trailing head (the transition shaded in light green). The trailing ADP-head then diffuses to the INT position with probability *P*_0_ (Figure 1b), as just mentioned above. This INT (or one-head-bound) state (Figure 1b) is consistent with the available cryo-EM image [52] and recent all-atom MD simulations (Shi et al., to be published in *Proteins*) showing that one head in ϕ state binds to MT and the other head is in the above position of the MT-bound head. After ATP binding to the MT-bound head, NL docking takes place, weakening the interaction between the two heads (Figure 1c). Then, by overcoming the weak binding energy (*E*_I_) between the two heads and the energy change ($\Delta E_{\mathrm{NL}}$) arising from the stretching of the NLs, the ADP-head (with a probability *P*_E_) diffuses to and binds to the nearest front binding site IV on MT with affinity *E*_w2_, and ADP is released (Figure 1d). From Figure 1a to b to c to d, a forward step was made by hydrolyzing an ATP molecule. From Figure 1c, it is also possible (with probability 1–*P*_E_) that by overcoming *E*_I_, NL-docking energy (*E*_D_) of the MT-bound head and $\Delta E_{\mathrm{NL}}$, the ADP-head diffuses to and binds to the previous site II with affinity *E*_w2_ (noting that after Pi release the binding energy of site II for ADP-head changes rapidly from *E*_w1_ to *E*_w2_ in time *t*_r_ in the order of μs), and ADP is released (Figure 1e). The transition (from Figure 1a to b to c to e) gives a futile chemomechanical coupling cycle.

In Figure 1e after the ATPase activity (ATP hydrolysis and then Pi release) taking place in the leading head, there are also two possible cases that can occur. There is a probability 1–*P*_0_ that the head still binds to site III but an ATPase cycle (consisting of ADP release, ATP binding, ATP hydrolysis, and then Pi release) takes place (the transition shaded in light green). There is also a probability *P*_0_ that the ADP-head diffuses to the INT position (Figure 1f). ATP binding to the MT-bound head induces its NL docking, weakening its interaction with the ADP-head (Figure 1g). Then, by overcoming *E*_I_ and $\Delta E_{\mathrm{NL}}$ the ADP-head rebinds to the previous site III with affinity *E*_w2_, and releases ADP (Figure 1a). The transition (from Figure 1e to f to g to a) gives a futile chemomechanical coupling cycle. From Figure 1g, it is also possible that by overcoming *E*_I_, *E*_D_, and $\Delta E_{\mathrm{NL}}$ the ADP-head diffuses to and binds to site I with affinity *E*_w2_, and releases ADP (Figure 1h). The transition (from Figure 1e to f to g to h) gives a backward step by hydrolyzing one ATP molecule. Because the transitions of Figure 1g to a and to h overcome the same energy barriers as the transitions of Figure 1c to d and to e, respectively, the transition from Figure 1g to a and that from Figure 1c to d occur with the same probability *P*_E_ and the transition from Figure 1g to h and that from Figure 1c to e occur with the same probability 1–*P*_E_.

It should be noted that besides the above-mentioned transitions, other possible transitions can also occur, which are not drawn in Figure 1. For example, in Figure 1e before ATP hydrolysis occurs in the leading head ATP can bind to the trailing head, becoming the state with both heads bound by ATP. Similarly, in Figure 1a before ATP hydrolysis occurs in the trailing head ATP can bind to the leading head, also becoming the state with both heads bound by ATP. From the state with both heads in ATP state, if the ATPase activity (ATP hydrolysis and then Pi release) takes place in the trailing head the system becomes the state of Figure 1c with probability *P*_0_, while if the ATPase activity takes place in the leading head the system becomes the state of Figure 1g with probability *P*_0_.

As all-atom MD simulations showed [39,40,53], for WT kinesin-1 with the NL of each head having 14 residues, when the two heads are bound to two successive binding sites on an MT filament that are 8.2-nm apart the internally stretched force on the NLs has a large value (about 30 pN). Driven by the large internally stretched force and the thermal noise, the ADP-head can escape from the potential well of depth *E*_w1_ within the time period *t*_r_ with nearly 100% probability [39,40]. Thus, for a good approximation, we take *P*_0_ = 1. That is, the transitions shaded in light green cannot occur, with the pathway becoming identical to that presented before [38]. By contrast, for mutant kinesin-1 with each NL being extended by six residues, when the two heads are bound to two successive binding sites on an MT filament that are 8.2-nm apart the internally stretched force in the NLs is nearly zero [39,40]. Thus, after Pi release, the ADP-head may not escape from the potential well of depth *E*_w1_ within the time period *t*_r_ with 100% probability, i.e., with *P*_0_ < 1.

As done in the single-molecule optical trapping assays [15,16,17,18,19,20,21,22,23,24,25,26], we consider a bead attached to the coiled-coil stalk that connects the NLs of the two heads and an external force, *F*, acting on the bead. The force *F* is defined to be positive when pointing backward. The available experimental data showed that extending the NLs of the kinesin-1 dimer by any length via inserting additional residues into the NLs has nearly no effect on the ATPase rate of the dimer during its processive movement on MT [27]. Since changing the length of the NLs changes the strain on the NLs when the two heads are bound to MT, the experimental data thus imply that the strain on the NL has no effect on the ATPase rate (at least the rate constants of the rate-limiting steps of the ATPase activity such as ATP hydrolysis and Pi release) of the kinesin head. Consequently, we propose that changing the NL length has no effect on the ATPase rate, and the external force *F*, which affects the strain on the NL, also has no effect on the ATPase rate. However, the orientation direction of the NL has an effect on the ATPase rate: the trailing head with the forward NL orientation has a much higher ATPase rate than the leading head with the backward NL orientation. This can be understood as follows. The NL with the forward orientation can interact with the head and the interaction enhances the ATPase rate, and by contrast, the NL with the backward orientation has no interaction with the head. This is consistent with the experimental data showing that by deleting the NL, equivalent to removing the interaction of the NL with the head, the ATPase rate is reduced greatly [54].

### 2.2. The Simplified Model at Saturating ATP

**Figure 2:**
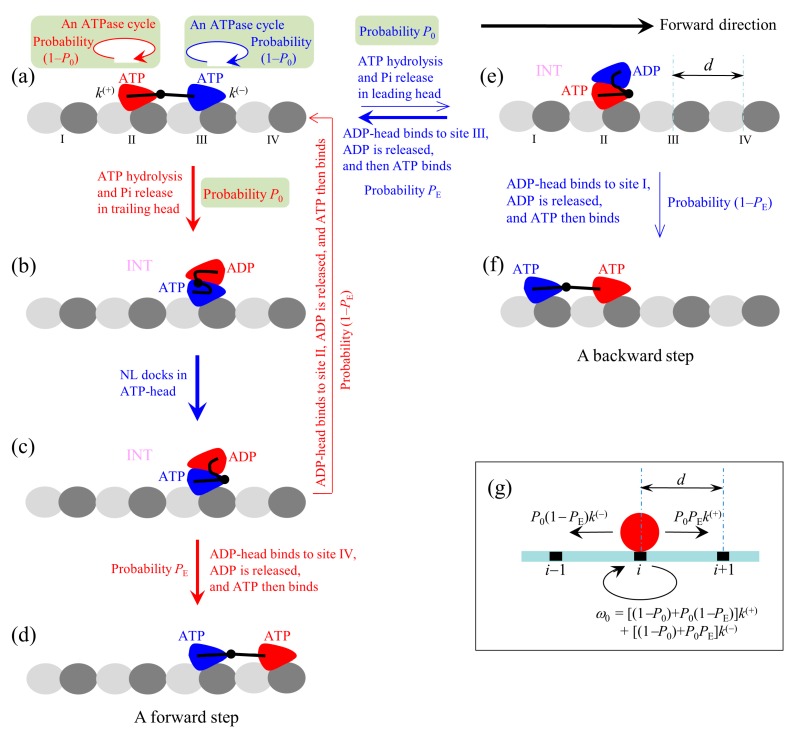
The model of stepping of kinesin dimer at saturating ATP. (**a**–**f**) The pathway for the chemomechanical coupling (see text for detailed description). The thickness of the arrow represents the magnitude of the transition probability under no external force. (**g**) The simplified model derived from the pathway shown in (**a**–**f**). The red circle represents the center of mass of the motor. The positions of binding sites on the MT filament are denoted by …, (*i*–1), *i*, (*i*+1), ….

At saturating ATP, the chemomechanical coupling pathway shown in Figure 1 can be simplified to that shown in Figure 2a–f. We begin with both heads in ATP state binding strongly to MT (Figure 2a).

First, consider an ATPase activity (ATP hydrolysis and then Pi release) taking place in the trailing head. Then, two possible cases can occur, as discussed above. One case, with probability 1–*P*_0_, is that the head still binds to site II but an ATPase cycle (consisting of ADP release, ATP binding, ATP hydrolysis and Pi release) takes place in the head (the transition shaded in light green). Another case, with probability *P*_0_, is that the ADP-head diffuses to the INT position relative to the MT-bound head (Figure 2b). In the INT state, with the MT-bound head in ATP state, NL docking takes place, weakening the interaction between the two heads (Figure 2c). Then, the ADP-head (with probability *P*_E_) diffuses to and binds to the nearest front site IV with affinity *E*_w2_, and ADP is released, which is followed immediately by ATP binding (Figure 2d). From Figure 2a to d, a forward step was made by hydrolyzing an ATP molecule. From Figure 2c, it is also possible (with probability 1–*P*_E_) that the ADP-head diffuses to and binds to the previous site II with affinity *E*_w2_, and ADP is released, which is followed immediately by ATP binding (Figure 2a). The transition (from Figure 2a to c and then returning to a) gives a futile chemomechanical coupling cycle.

Second, consider in Figure 2a an ATPase activity (ATP hydrolysis and then Pi release) taking place in the leading head. Two possible cases can occur. One case, with probability 1–*P*_0_, is that the head still binds to site III but an ATPase cycle (consisting of ADP release, ATP binding, ATP hydrolysis and Pi release) takes place in the head (the transition shaded in light green). Another case, with probability *P*_0_, is that the ADP-head diffuses to the INT position (Figure 2e). After the binding affinity of site III for ADP-head changes rapidly back to *E*_w2_ in time *t*_r_, the detached ADP-head rebinds to site III, with probability *P*_E_, and ADP is released, followed immediately by ATP binding (Figure 2a). The transition (from Figure 2a to e and then returning to a) gives a futile chemomechanical coupling cycle. From Figure 2e, it is also possible that the ADP-head diffuses to and binds to site I with affinity *E*_w2_, with probability 1–*P*_E_, and ADP is released, followed immediately by ATP binding (Figure 2f). The transition (from Figure 2a to e to f) gives a backward step by hydrolyzing an ATP molecule.

The pathway (Figure 2a–f) can be simply described as follows. We denote by *k*^(+)^ and *k*^(^^−^^)^ the rate constants of ATP hydrolysis and Pi release in the trailing and leading heads at saturating ATP, respectively. Considering that the step of ATP hydrolysis and Pi release is rate limiting in the ATPase activity at saturating ATP, it is approximate that *k*^(+)^ and *k*^(^^−^^)^ are ATPase rates of the trailing and leading heads, respectively. Since under physiological conditions an ATP hydrolysis gives free energy ≈20 *k*_B_*T* and *k*^(+)^ and *k*^(^^−)^ are constants independent of the NL strain and external force (see above), it is a good approximation to consider the transitions of the ATPase activity to be irreversible under any external force, i.e., to ignore the reverse transitions of the ATPase activity. Since in the INT state with the MT-head bound with ATP (Figure 2b) its NL docks rapidly [55], we approximate that the NL docking occurs immediately. Thus, during processive movement of the dimer the total ATPase rate is *k*^(+)^ + *k*^(^^−)^, a constant independent of the NL length and external force, which is consistent with the available experimental data [27]. When an ATPase activity with rate constant *k*^(+)^ takes place, the motor either takes a forward step with probability *P*_0_*P*_E_ or does not move with probability (1–*P*_0_) + *P*_0_(1–*P*_E_). When an ATPase activity with rate constant *k*^(^^−)^ takes place, the motor either takes a backward step with probability *P*_0_(1–*P*_E_) or does not move with probability (1–*P*_0_) + *P*_0_*P*_E_. Since after detaching from MT the time for the ADP-head to diffuse from the binding site on MT to INT position and the time for the ADP-head to diffuse from the INT position to binding site on MT are much shorter than the inverse of the ATPase rates [39], for a good approximation, the velocity of the motor is determined solely by the ATPase rates of the two heads. Hence, the overall forward stepping rate of the motor is *P*_0_*P*_E_*k*^(+)^, backward stepping rate is *P*_0_(1–*P*_E_)*k*^(^^−)^, and ATPase rate with no stepping is *ω*_0_ = [(1–*P*_0_) + *P*_0_(1–*P*_E_)]*k*^(+)^ + [(1–*P*_0_) + *P*_0_*P*_E_]*k*^(^^−)^, as schematically shown in Figure 2g.

### 2.3. Dynamics of WT Kinesin-1 at Saturating ATP

For the case of WT kinesin dimer, *P*_0_ = 1 (see above) and the transitions shaded in light green in Figure 2 can be excluded, as studied in the previous work [38]. Since NL is flexible it is reasonable to consider that when only one head is bound to MT the interaction between the attached bead and kinesin dimer acts only on the NL of the head having a larger distance to the bead. Thus, when only one head is bound to MT, the backward external force (*F* > 0) acts only on the NL of the head in the leading position and the forward external force (*F* < 0) acts only on the NL of the head in the trailing position. As a result, after the trailing head detaches from site II, *F* > 0 has no effect on its movement to the INT position and *F* < 0 facilitates its movement to the INT position, giving the transition from Figure 2a to b with 100% probability for any *F*. After the leading head detaches from site III, *F* > 0 facilitates its movement to the INT position and *F* < 0 has no effect on its movement to the INT position, giving the transition from Figure 2a to e with 100% probability for any *F*. When *F* > 0 the force dependence of the rate for the detached ADP-head to move from the INT position to the front binding site on MT has the form $k_{F}=C_{1}\exp\left(-\beta Fd^{\left(+\right)}\right)$ and the rate for the detached ADP-head to move from the INT position to the rear binding site on MT has the form $k_{R}=C_{1}\exp\left(-\beta E_{D}\right)$ (see Section S1 in Supplementary Information). Here, *C*_1_ is a constant independent of *F*, $\beta^{-1}=k_{B}T$, with *k*_B_ being the Boltzmann constant and *T* the absolute temperature, *d*^(+)^ is the characteristic distance for the movement of the detached ADP-head from the INT position to the front binding site on MT, and *E*_D_ is the NL-docking energy and. Throughout we consider room temperature (*T* = 298 K) unless otherwise pointed out.

With *k*_F_ and *k*_R_, probability *P*_E_ can be calculated by $P_{E}=k_{F}/\left(k_{F}+k_{R}\right)$ = $C_{1}\exp\left(-\beta Fd^{\left(+\right)}\right)/\left[C_{1}\exp\left(-\beta Fd^{\left(+\right)}\right)+C_{1}\exp\left(-\beta E_{D}\right)\right]$. Hence, the force dependence of the stepping ratio (defined as the ratio of the number of forward steps to that of backward steps) of the motor can be calculated by *r* = $P_{E}k^{\left(+\right)}/\left[\left(1-P_{E}\right)k^{\left(-\right)}\right]$ = $\left(k^{\left(+\right)}/k^{\left(-\right)}\right)\exp\left(\beta E_{D}\right)\exp\left(-\beta d^{\left(+\right)}F\right)$ = $r_{0}\exp\left(-\alpha^{\left(+\right)}F\right)$, where *r*_0_ = $\left(k^{\left(+\right)}/k^{\left(-\right)}\right)\exp\left(\beta E_{D}\right)$ is the stepping ratio under no external force and $\alpha^{\left(+\right)}=\beta d^{\left(+\right)}$ is independent of *F*. Similarly, when *F* < 0 the force dependence of the stepping ratio has the form, $r=r_{0}\exp\left(-\alpha^{\left(-\right)}F\right)$, where $\alpha^{\left(-\right)}=\beta d^{\left(-\right)}$ is independent of *F*, with *d*^(^^−^^)^ being the characteristic distance for the movement of the detached ADP-head from the INT position to the rear binding site on MT. For approximation, $d^{\left(+\right)}=d^{\left(-\right)}$ and $\alpha^{\left(+\right)}=\alpha^{\left(-\right)}=\alpha$. Therefore, for both *F* > 0 and *F* < 0, the force dependence of the stepping ratio can be re-expressed as

(1) $$r=r_{0}{}^{\left(1-F/F_{S}\right)}$$

(2) $$r_{0}=\left(k^{\left(+\right)}/k^{\left(-\right)}\right)\exp\left(\beta E_{D}\right)$$

where $F_{S}=\ln\left(r_{0}\right)/\alpha$ is the stall force, at which *r* = 1, and *E*_D_ is the NL-docking energy (see above). Using Equation (1) and relation $r=P_{E}k^{\left(+\right)}/\left[\left(1-P_{E}\right)k^{\left(-\right)}\right]$, probability *P*_E_ can be re-expressed as

(3) $$P_{E}=\frac{r_{0}{}^{\left(1-F/F_{S}\right)}}{r_{0}{}^{\left(1-F/F_{S}\right)}+k^{\left(+\right)}/k^{\left(-\right)}}$$

From Figure 2g with *P*_0_ = 1, the velocity of the motor can be expressed as

(4) $$v=\left[P_{E}k^{\left(+\right)}-\left(1-P_{E}\right)k^{\left(-\right)}\right]d$$

where *d* = 8.2 nm is the step size equal to the period of the MT filament. Substituting Equation (3) into Equation (4) we obtain

(5) $$v=\frac{r_{0}{}^{\left(1-F/F_{S}\right)}-1}{r_{0}{}^{\left(1-F/F_{S}\right)}+k^{\left(+\right)}/k^{\left(-\right)}}k^{\left(+\right)}d$$

We then derive the equation for mean dwell time between two mechanical steps. If *n* (*n* > 0 is an integer) ATP molecules are hydrolyzed for the motor to make a mechanical step (either a forward or backward step), the dwell time before stepping is $n/\left(k^{\left(+\right)}+k^{\left(-\right)}\right)$. The probability for the motor to make a mechanical step after hydrolyzing *n* ATP molecules can be expressed as $\left[k^{\left(+\right)}P_{E}+k^{\left(-\right)}\left(1-P_{E}\right)\right]{\left[k^{\left(+\right)}\left(1-P_{E}\right)+k^{\left(-\right)}P_{E}\right]}^{n-1}/{\left(k^{\left(+\right)}+k^{\left(-\right)}\right)}^{n}$. Hence, the mean dwell time between two mechanical steps can be calculated by

(6) $$T_{d}=\sum_{n=1}^{\infty }\left\{\frac{n}{k^{\left(+\right)}+k^{\left(-\right)}}\frac{\left[k^{\left(+\right)}P_{E}+k^{\left(-\right)}\left(1-P_{E}\right)\right]{\left[k^{\left(+\right)}\left(1-P_{E}\right)+k^{\left(-\right)}P_{E}\right]}^{n-1}}{{\left(k^{\left(+\right)}+k^{\left(-\right)}\right)}^{n}}\right\}$$

Equation (6) can be reduced as $T_{d}=1/\left[k^{\left(+\right)}P_{E}+k^{\left(-\right)}\left(1-P_{E}\right)\right]$, which with Equation (3) can be re-expressed as

(7) $$T_{d}=\frac{r_{0}{}^{\left(1-F/F_{S}\right)}+k^{\left(+\right)}/k^{\left(-\right)}}{r_{0}{}^{\left(1-F/F_{S}\right)}+1}\frac{1}{k^{\left(+\right)}}$$

**Figure 3:**
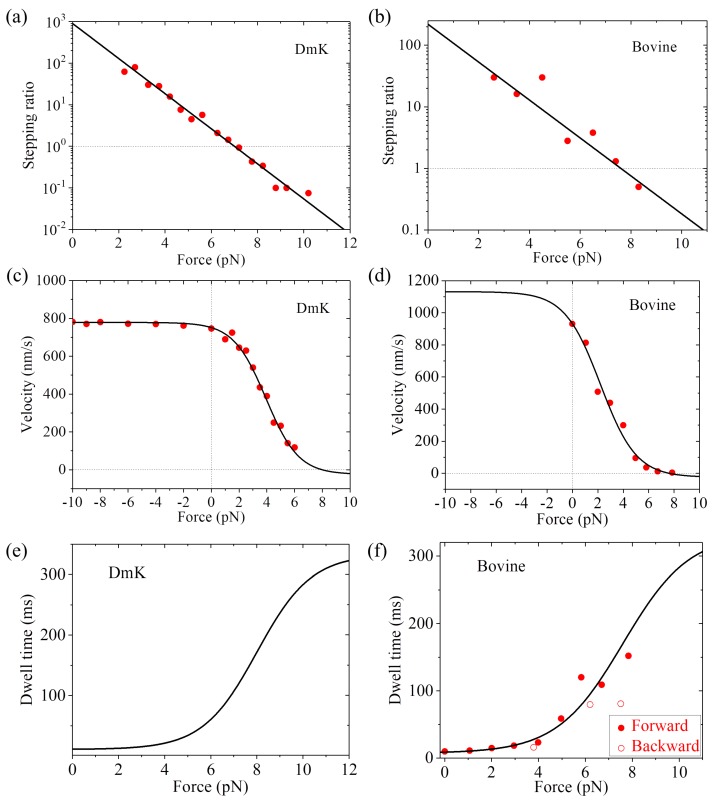
Dynamics of wild type (WT) kinesin-1 at saturating ATP. (**a**) Stepping ratio versus external force for DmK. Line represents analytical solution. Symbols are experimental data at 1 mM ATP taken from Carter and Cross [23]. Adapted by permission from Springer Nature: Carter, N.J.; Cross, R.A. Mechanics of the kinesin step. *Nature* **2005**, *435*, 308–312. Licence number: 4680770608424 (**b**) Stepping ratio versus external force for bovine brain kinesin (Bovine). Line represents analytical solution. Symbols are experimental data at 1 mM ATP taken from Nishiyama et al. [20]. Adapted by permission from Springer Nature: Nishiyama, M.; Higuchi, H.; Yanagida, T. Chemomechanical coupling of the forward and backward steps of single kinesin molecules. *Nat. Cell Biol.* **2002**, *4*, 790–797. Licence number: 4680771132891. (**c**) Velocity versus external force for DmK. Line represents analytical solution. Symbols are experimental data at 2 mM ATP taken from Andreasson et al. [24]. (**d**) Velocity versus external force for Bovine. Line represents analytical solution. Symbols are experimental data at 1 mM ATP taken from Nishiyama et al. [20]. Adapted by permission from Springer Nature: Nishiyama, M.; Higuchi, H.; Yanagida, T. Chemomechanical coupling of the forward and backward steps of single kinesin molecules. *Nat. Cell Biol.* **2002**, *4*, 790–797. Licence number: 4680771132891. (**e**) Dwell time versus external force for DmK. Line represents analytical solution. (**f**) Dwell time versus external force for Bovine. Line represents analytical solution. Symbols are experimental data at 1 mM ATP taken from Nishiyama et al. [20] Adapted by permission from Springer Nature: Nishiyama, M.; Higuchi, H.; Yanagida, T. Chemomechanical coupling of the forward and backward steps of single kinesin molecules. *Nat. Cell Biol.* **2002**, *4*, 790–797. Licence number: 4680771132891.

Using Equations (1), (5), and (7), the available single-molecule data on the force dependence of stepping ratio, velocity and dwell time at saturating ATP for WT kinesin-1 dimers can be reproduced quantitatively with only four adjustable parameters, *r*_0_, *F*_S_, *k*^(+)^ and *k*^(^^−)^. Each parameter has a clear physical meaning: *r*_0_ is the stepping ratio under no external force, *F*_S_ is the stall force, and *k*^(+)^ and *k*^(^^−)^ are the ATPase rates of the trailing and leading heads, respectively. Using Equation (1) and taking *r*_0_ = 900 and *F*_S_ = 7 pN, the single-molecule data of Carter and Cross [23] on the force dependence of stepping ratio for *Drosophila* kinesin (abbreviated as DmK) at saturating ATP (1 mM) can be reproduced (Figure 3a). Adjusting *r*_0_ = 220 and *F*_S_ = 7.6 pN, the single-molecule data of Nishiyama et al. [20] on the force dependence of stepping ratio for bovine brain kinesin (abbreviated as Bovine) at saturating ATP (1 mM) can be reproduced (Figure 3b). Using Equation (5), the single-molecule data of Andreasson et al. [24] on the force dependence of velocity for DmK at saturating ATP (2 mM) can be fitted well by adjusting *r*_0_ = 890, *F*_S_ = 8 pN, *k*^(+)^ = 95 $s^{-1}$ and *k*^(^^−)^ = 3 $s^{-1}$ (see Table 1, Figure 3c). Note here that *r*_0_ = 890 is close to that used in Figure 3a for DmK, and *F*_S_ = 8 pN is slightly larger than that used in Figure 3a. The slight difference of *F*_S_ could be due to different buffer conditions in different experiments [23,24]. With *r*_0_ = 220 and *F*_S_ = 7.6 pN (see Figure 3b) and adjusting *k*^(+)^ = 138 $s^{-1}$ and *k*^(^^−)^ = 3 $s^{-1}$ (see Table 1), the single-molecule data of Nishiyama et al. [20] on the force dependence of velocity for Bovine at saturating ATP (1 mM) can be fitted well (Figure 3d). Using Equation (7) and with parameter values given in Table 1 for DmK, the theoretical results of dwell time versus force are shown in Figure 3e. Using Equation (7) and with parameter values given in Table 1 for Bovine, the theoretical results of dwell time versus force are in agreement with the single-molecule data of Nishiyama et al. [20] at saturating ATP (1 mM) (Figure 3f).

More interestingly, with above fitted parameter values and Equation (2) we can determine the NL-docking energy *E*_D_ and compare with the available experimental data [56]. With *r*_0_ = 890 ± 120, *k*^(+)^ = 95 ± 1 $s^{-1}$ and *k*^(^^−)^ = 3 ± 0.4 $s^{-1}$ for DmK (see Table 1), we obtain *E*_D_ = (3.34 ± 0.28) *k*_B_*T*. Here, from Equation (2), the error of *E*_D_ is calculated with $\Delta E_{D}$ = $\left|\partial E_{D}/\partial r_{0}\right|\Delta r_{0}+\left|\partial E_{D}/\partial k^{\left(-\right)}\right|\Delta k^{\left(-\right)}+\left|\partial E_{D}/\partial k^{\left(+\right)}\right|\Delta k^{\left(+\right)}$ = $\beta^{-1}\left(\Delta r_{0}/r_{0}+\Delta k^{\left(-\right)}/k^{\left(-\right)}+\Delta k^{\left(+\right)}/k^{\left(+\right)}\right)$, where $\Delta r_{0}$, $\Delta k^{\left(-\right)}$ and $\Delta k^{\left(+\right)}$ represent errors of *r*_0_, *k*^(^^−)^ and *k*^(+)^, respectively. With *r*_0_ = 220 ± 68, *k*^(+)^ = 138 ± 20 $s^{-1}$ and *k*^(−)^ = 3 ± 1.1 $s^{-1}$ for Bovine (see Table 1), we obtain *E*_D_ = (1.56 ± 0.82) *k*_B_*T*. These small values of *E*_D_ = (3.34 ± 0.28) *k*_B_*T* and (1.56 ± 0.82) *k*_B_*T* are close to the available experimental data showing that the free energy change associated with the NL docking is only about (1 — 2) *k*_B_*T* [56]. It is noted that in the prevailing models presented in the literature proposing that the NL docking drives the forward movement of the ADP-head, a persistent problem is that the small NL-docking energy of (1 — 2) *k*_B_*T* is only about 10% of the energy required to generate an 8.2-nm step against a load of 7 — 8 pN [57,58]. Here, with our model proposing that the NL docking provides an energy barrier to prevent the detached ADP-head in the INT position from moving backward and the ATPase rates of the two heads are independent of *F*, this puzzling issue can be understandable easily. In addition, from the fitted curve in Figure 3a we have *d*^(+)^ = 4 nm, which is understandable easily by considering that *d*^(+)^ should be smaller than or about half of *d* = 8.2 nm. By contrast, with the prevailing models presented in the literature proposing that the force dependence of velocity is determined by the force dependence of ATPase rate, it is puzzling why the related characteristic distance is only about half of the movement distance of the motor in one step [28].

**Figure 4:**
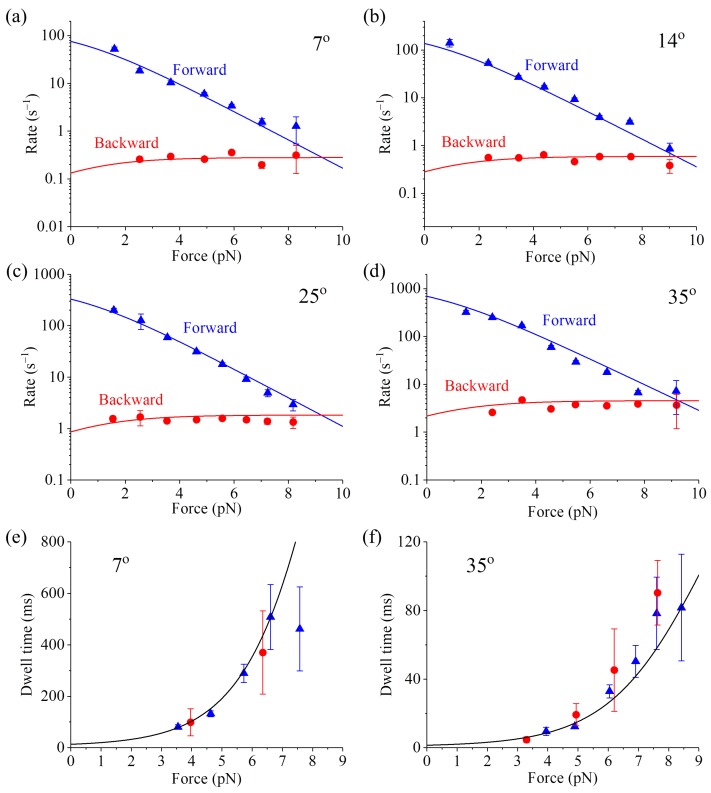
Thermodynamics of WT bovine brain kinesin-1 at saturating ATP. Lines represent analytical solutions. Symbols are experimental data at 1 mM ATP taken from Taniguchi et al. [59]. Adapted by permission from Springer Nature: Taniguchi, Y.; Nishiyama, M.; Ishii, Y.; Yanagida, T. Entropy rectifies the Brownian steps of kinesin. *Nat. Chem. Biol.* **2005**, *1*, 342–347. Licence number: 4680771382349. (**a**–**d**) Forward and backward stepping rates versus external force at different temperatures. (**e**,**f**) Dwell times between two mechanical steps versus external force at different temperatures. Blue and red symbols represent the experimentally measured dwell times for forward and backward steps, respectively.

In addition, other available single-molecule data, such as those on the dependence of the forward stepping rate, backward stepping rate and dwell time upon backward force (*F* > 0) and temperature (*T*) at saturating ATP [59], can also be reproduced quantitatively (Figure 4, see Section S2 in Supplementary Information for related equations). As shown in Table S1 (see Supplementary Information), only four parameters are adjusted to fit all the single-molecule data (10 curves) shown in Figure 4. Interestingly, the fitted value of *E*_D_ = 0.4 pN nm (see Table S1) implies that the free energy change of NL docking is very small, also consistent with the experimental result [56]. The analytical result of the stall force being independent of temperature (see Equation (S15) in Section S2) is also in agreement with the available single-molecule data [60].

It is mentioned that in the calculation here, we use the step size of a constant value of *d* = 8.2 nm, which is equal to the periodicity of an MT filament. However, from the published experimental results, the step size fluctuated in a range of ±2 nm. The fluctuations of the step size could be due to the large fluctuations of the position of the attached bead which was detected in the experiments. Another point to note here is that for simplicity of analysis, we have not considered the effect of the length of the coiled-coil stalk on kinesin movement. With a model system simulating complex of kinesin dimer and MT, it was demonstrated that the interaction between the stalk and MT provides the force to drive the motion of the motor [61]. Using single-molecule optical trapping, Asbury et al. [21] found that WT kinesin-1 homodimer moves in an asymmetric hand-over-hand manner (called limping), and the length of the stalk affects the limping character. Although the effect of the stalk length on the limping effect of kinesin homodimers has been studied numerically based on our model (Figure 2) [40], it is interesting to have an analytical study on the effect of the stalk length on the kinesin movement, which will be studied in the future.

### 2.4. Dynamics of Kinesin-1 with Extended NLs at Saturating ATP

In the above section, we studied analytically the dynamics of WT kinesin-1 dimer, with *P*_0_ = 1. In this section, we will study analytically the dynamics of mutant kinesin-1 dimer of extended NLs. For example, we consider that the NL is extended by inserting six additional residues AEQKLT into the C-terminal portion of the NL region of each head, as done in Clancy et al. [26]. As all-atom MD simulations showed [39,40], for the mutant kinesin dimer with the extended NLs, when the two heads are bound to two successive binding sites on an MT filament the internally stretched force between the NLs is nearly equal to zero. Thus, after Pi release the ADP-head may not escape from the potential well of depth *E*_w1_ within the time period *t*_r_ with 100% probability, implying *P*_0_ < 1 under no external force. In addition, since the NL is flexible and the internally stretched force is nearly equal to zero, the backward external force *F* (> 0) acts only on the leading head while the forward external force *F* (< 0) acts only on the trailing head. In the following we consider the backward force *F* > 0 and forward force *F* < 0, separately.

First, consider *F* > 0. Since *F* affects only the leading head while has no effect on the trailing head, at saturating ATP the forward stepping rate is $P_{0}(0)P_{E}(F)k^{\left(+\right)}$ and the backward stepping rate is $P_{0}(F)\left[1-P_{E}(F)\right]k^{\left(-\right)}$ (see Figure 2g), where *P*_0_(0) is the probability for the kinesin head to escape from the potential well of depth *E*_w1_ within the time period *t*_r_ under *F* = 0. For approximation, the force dependence of *P*_0_(*F*) can be calculated by

(8) $$P_{0}(F)=1-\left[1-P_{0}(0)\right]\exp\left(-\beta F\delta \right)\;\;\mathrm{for}F\leq 0$$

where δ is the characteristic distance, which should be equal to the Debye length of about 1 nm in solution, and thus we take δ = 1 nm.

Probability *P*_E_ can still be calculated by *P*_E_(*F*) = $k_{F}/\left(k_{F}+k_{R}\right)$ = $C_{1}\exp\left(-\beta Fd^{\left(+\right)}\right)/\left[C_{1}\exp\left(-\beta Fd^{\left(+\right)}\right)+C_{1}\exp\left(-\beta E_{D}\right)\right]$, where *C*_1_ has the same form as that for the WT case except that the energy change $\Delta E_{\mathrm{NL}}$ arising from NL stretching has a different value from that for the WT case (see Section S1). As it is seen, *P*_E_(*F*) is independent of *C*_1_. Thus, the above equation for *P*_E_(*F*) is the same as that for the WT case, which can also be written as Equation (3).

The stepping ratio can be calculated by $r=P_{0}(0)P_{E}(F)k^{\left(+\right)}/\left\{P_{0}(F)\left[1-P_{E}(F)\right]k^{\left(-\right)}\right\}$. Substitution of Equation (3) into above equation yields

(9) $$r=r_{0}{}^{\left(1-F/F_{S}\right)}\frac{P_{0}(0)}{P_{0}(F)}$$

Note that for the WT case, *P*_0_(*F*) = *P*_0_(0) = 1 and Equation (9) becomes Equation (1). It should be emphasized that in Equation (9) only under *P*_0_(0) = *P*_0_(*F*) the stall force is equal to *F*_S_, whereas under *P*_0_(0) ≠ *P*_0_(*F*) the stall force is no longer equal to *F*_S_. In other words, *F*_S_ represents the stall force only under case of *P*_0_(*F*) = *P*_0_(0) = 1 (i.e., the WT case). By contrast, *r*_0_ is the stepping ratio at *F* = 0 under any case.

The velocity can be calculated by $v=\left\{P_{0}(0)P_{E}(F)k^{\left(+\right)}-P_{0}(F)\left[1-P_{E}(F)\right]k^{\left(-\right)}\right\}d$. Substitution of Equation (3) into above equation yields

(10) $$v=\frac{P_{0}(0)r_{0}{}^{\left(1-F/F_{S}\right)}-P_{0}(F)}{r_{0}{}^{\left(1-F/F_{S}\right)}+k^{\left(+\right)}/k^{\left(-\right)}}k^{\left(+\right)}d$$

Note that for the WT case, *P*_0_(*F*) = *P*_0_(0) = 1 and Equation (10) becomes Equation (4).

The diffusion constant is calculated by $D=\underset{t\to \infty }{\lim}\left[\langle x^{2}(t)\rangle -{\langle x(t)\rangle }^{2}\right]/\left(2t\right)$, where *x* is the center-of-mass position of the motor along the movement direction in a movement trace. From this equation, we finally obtain $D=\left\{P_{0}(0)P_{E}(F)k^{\left(+\right)}+P_{0}(F)\left[1-P_{E}(F)\right]k^{\left(-\right)}\right\}d^{2}/2$. Substitution of Equation (3) into above equation yields

(11) $$D=\frac{1}{2}\frac{P_{0}(0)r_{0}{}^{\left(1-F/F_{S}\right)}+P_{0}(F)}{r_{0}{}^{\left(1-F/F_{S}\right)}+k^{\left(+\right)}/k^{\left(-\right)}}k^{\left(+\right)}d^{2}$$

The randomness parameter, which is defined as $R=\underset{t\to \infty }{\lim}\left\{\left[\langle x^{2}(t)\rangle -{\langle x(t)\rangle }^{2}\right]/\left[d\langle x(t)\rangle \right]\right\}$, can be calculated by R=2D/(vd). Substitution of Equations (10) and (11) into above equation yields

(12) $$R=\frac{P_{0}(0)r_{0}{}^{\left(1-F/F_{S}\right)}+P_{0}(F)}{P_{0}(0)r_{0}{}^{\left(1-F/F_{S}\right)}-P_{0}(F)}$$

The mean number of ATP molecules consumed per forward step can be calculated by $N_{F}=\left(k^{\left(+\right)}+k^{\left(-\right)}\right)/\left(P_{0}(0)P_{E}(F)k^{\left(+\right)}\right)$. Substitution of Equation (3) into above equation yields

(13) $$N_{F}=\left(1+\frac{k^{\left(-\right)}}{k^{\left(+\right)}}\right)\frac{r_{0}{}^{\left(1-F/F_{S}\right)}+k^{\left(+\right)}/k^{\left(-\right)}}{P_{0}(0)r_{0}{}^{\left(1-F/F_{S}\right)}}$$

The mean number of ATP molecules consumed per step (either a forward or a backward step) can be calculated by $N=\left(k^{\left(+\right)}+k^{\left(-\right)}\right)/\left\{P_{0}(0)P_{E}(F)k^{\left(+\right)}+P_{0}(F)\left[1-P_{E}(F)\right]k^{\left(-\right)}\right\}$. Substitution of Equation (3) into above equation yields

(14) $$N=\left(1+\frac{k^{\left(-\right)}}{k^{\left(+\right)}}\right)\frac{r_{0}{}^{\left(1-F/F_{S}\right)}+k^{\left(+\right)}/k^{\left(-\right)}}{P_{0}(0)r_{0}{}^{\left(1-F/F_{S}\right)}+P_{0}(F)}$$

Second, consider *F* < 0, which affects only the trailing head while has no effect on the leading head. Similar to Equation (8) for *F* > 0, the force dependence of *P*_0_(*F*) for *F* < 0 can be calculated by

(15) $$P_{0}(F)=1-\left[1-P_{0}(0)\right]\exp\left(\beta F\delta \right)\;\;\mathrm{for}\;F\leq 0$$

Similar to the derivation of Equations (9)–(14) for *F* > 0, we can derive following equations for *F* < 0. The stepping ratio has the form

(16) $$r=r_{0}{}^{\left(1-F/F_{S}\right)}\frac{P_{0}(F)}{P_{0}(0)}$$

The velocity has the form

(17) $$v=\frac{P_{0}(F)r_{0}{}^{\left(1-F/F_{S}\right)}-P_{0}(0)}{r_{0}{}^{\left(1-F/F_{S}\right)}+k^{\left(+\right)}/k^{\left(-\right)}}k^{\left(+\right)}d$$

The diffusion constant has the form

(18) $$D=\frac{1}{2}\frac{P_{0}(F)r_{0}{}^{\left(1-F/F_{S}\right)}+P_{0}(0)}{r_{0}{}^{\left(1-F/F_{S}\right)}+k^{\left(+\right)}/k^{\left(-\right)}}k^{\left(+\right)}d^{2}$$

The randomness parameter has the form

(19) $$R=\frac{P_{0}(F)r_{0}{}^{\left(1-F/F_{S}\right)}+P_{0}(0)}{P_{0}(F)r_{0}{}^{\left(1-F/F_{S}\right)}-P_{0}(0)}$$

The mean number of ATP molecules consumed per forward step has the form

(20) $$N_{F}=\left(1+\frac{k^{\left(-\right)}}{k^{\left(+\right)}}\right)\frac{r_{0}{}^{\left(1-F/F_{S}\right)}+k^{\left(+\right)}/k^{\left(-\right)}}{P_{0}(F)r_{0}{}^{\left(1-F/F_{S}\right)}}$$

The mean number of ATP molecules consumed per step (either a forward or a backward step) has the form

(21) $$N=\left(1+\frac{k^{\left(-\right)}}{k^{\left(+\right)}}\right)\frac{r_{0}{}^{\left(1-F/F_{S}\right)}+k^{\left(+\right)}/k^{\left(-\right)}}{P_{0}(F)r_{0}{}^{\left(1-F/F_{S}\right)}+P_{0}(0)}$$

**Figure 5:**
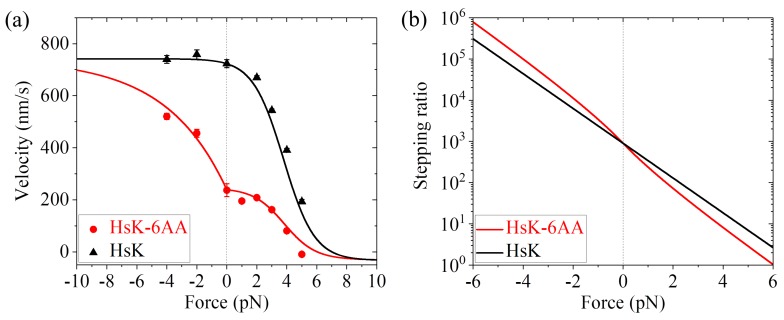
Dynamics of HsK and HsK-6AA at saturating ATP. (**a**) Velocity versus external force. Lines represent analytical solutions. Symbols are experimental data at 2 mM ATP taken from Andreasson et al. [24]. (**b**) Analytical solutions of stepping ratio versus external force.

Now, we can use Equations (8)–(21) to calculate velocity, stepping ratio, randomness parameter, ATP number per step, etc., at saturating ATP. First, we consider WT human kinesin-1 (abbreviated as HsK) and the one with its two NLs being extended by inserting six additional amino acids AEQKLT into the C-terminal portion of the NL region of each head (abbreviated as HsK-6AA). In our model, although the extension of the NLs for HsK-6AA changes the strain on the NLs, it has no effect on the ATPase activity of the head, the NL docking of the MT-bound head and the interaction of the head with MT. In addition, the extension has the same effect on the movement of the detached ADP-head from the INT position to the front binding site on MT and to the rear binding site on MT, thus having no or little effect on *r*_0_ and *F*_S_. Hence, we have the same values of *r*_0_, *F*_S_, *k*^(+)^, and *k*^(^^−)^ for both HsK and HsK-6AA. As mentioned above, *P*_0_ = 1 for HsK and *P*_0_ < 1 for HsK-6AA under *F* = 0. By taking *r*_0_ = 900 and *F*_S_ = 7 pN, as done in Figure 3a for DmK, and adjusting *k*^(+)^ = 90.5 $s^{-1}$, *k*^(^^−)^ = 4 $s^{-1}$, and *P*_0_(0) = 0.33 (see Table 1), the single-molecule data of Andreasson et al. [24] on the force dependence of velocity for both HsK and HsK-6AA at saturating ATP (2 mM) can be fitted well (Figure 5a). In other words, with the same four parameters *r*_0_, *F*_S_, *k*^(+)^, and *k*^(^^−)^ for both HsK and HsK-6AA and an additional parameter *P*_0_(0) for HsK-6AA, the calculated results for both HsK and HsK-6AA shown in Figure 5a are in quantitative agreement with the single-molecule data. The calculated results of the stepping ratio versus external force for HsK and HsK-6AA at saturating ATP are shown in Figure 5b. As expected, the stepping ratio for HsK-6AA at a given backward force (*F* > 0) is smaller than that for HsK whereas at a given forward force (*F* < 0) is larger than that for HsK.

**Figure 6:**
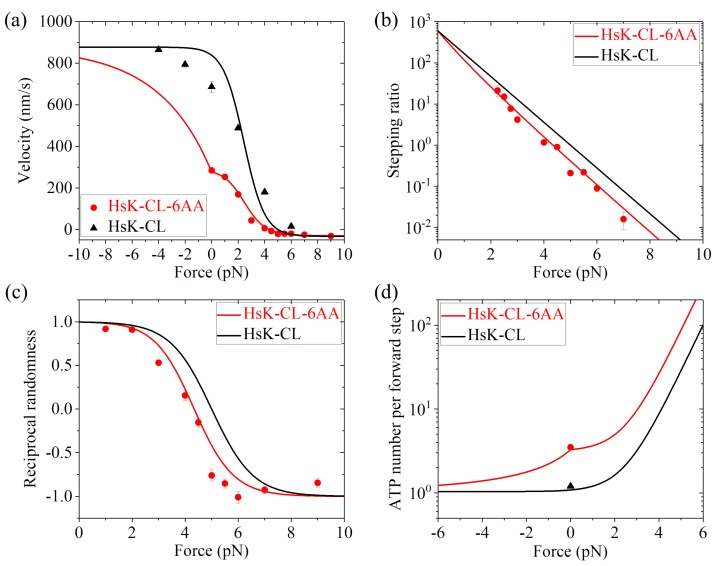
Dynamics of HsK-CL and HsK-CL-6AA at saturating ATP. (**a**) Velocity versus external force. Lines represent analytical solutions. Circles are experimental data for HsK-CL-6AA at 2 mM ATP taken from Clancy et al. [26] (adapted by permission from Springer Nature: Clancy, B.E.; Behnke-Parks, W.M.; Andreasson, J.O.L.; Rosenfeld, S.S.; Block, S.M. A universal pathway for kinesin stepping. *Nat. Struct. Mol. Biol.* **2011**, *18*, 1020–1027. Licence number: 4680780256064), while triangles are experimental data for HsK-CL at 2 mM ATP taken from Andreasson et al. [24]. (**b**) Stepping ratio versus external force. Lines represent analytical solutions. Symbols are experimental data for HsK-CL-6AA at 2 mM ATP taken from Clancy et al. [26]. Adapted by permission from Springer Nature: Clancy, B.E.; Behnke-Parks, W.M.; Andreasson, J.O.L.; Rosenfeld, S.S.; Block, S.M. A universal pathway for kinesin stepping. *Nat. Struct. Mol. Biol.* **2011**, *18*, 1020–1027. Licence number: 4680780256064. (**c**) Reciprocal randomness versus external force. Lines represent analytical solutions. Symbols are experimental data for HsK-CL-6AA at 2 mM ATP taken from Clancy et al. [26]. Adapted by permission from Springer Nature: Clancy, B.E.; Behnke-Parks, W.M.; Andreasson, J.O.L.; Rosenfeld, S.S.; Block, S.M. A universal pathway for kinesin stepping. *Nat. Struct. Mol. Biol.* **2011**, *18*, 1020–1027. Licence number: 4680780256064. (**d**) Number of ATP molecules consumed per forward step versus external force. Lines represent analytical solutions. Circle is the experimental data for HsK-CL-6AA at 2 mM ATP taken from Clancy et al. [26] (adapted by permission from Springer Nature: Clancy, B.E.; Behnke-Parks, W.M.; Andreasson, J.O.L.; Rosenfeld, S.S.; Block, S.M. A universal pathway for kinesin stepping. *Nat. Struct. Mol. Biol.* **2011**, *18*, 1020–1027. Licence number: 4680780256064), while triangle is the experimental data for HsK-CL at 1 mM ATP taken from Yildiz et al. [27].

Second, we consider human kinesin-1 with a residue cysteine in each of the two NL domains being mutated (abbreviated as HsK-CL) and the one with its two NLs being extended by inserting six additional residues AEQKLT into the C-terminal portion of the NL region of each head (abbreviated as HsK-CL-6AA). For the leading head with the backward orientation of its NL, no interaction would be present between the head and its NL. Thus, the mutation of a residue cysteine in the NL domain (HsK-CL and HsK-CL-6AA) would have no effect on the ATPase activity of the leading head and the interaction of the leading head with MT. Consequently, value of *k*^(^^−)^ and value of *P*_0_(0) for HsK-CL and HsK-CL-6AA should be equal to those for HsK and HsK-6AA, respectively. As a result, we still take *k*^(^^−)^ = 4 $s^{-1}$ for HsK-CL and HsK-CL-6AA, *P*_0_(0) = 1 for HsK-CL, and *P*_0_(0) = 0.33 for HsK-CL-6AA, same as those for HsK and HsK-6AA. However, since the mutation of the NL would affect the NL docking and the interaction between the NL and the trailing head with the forward orientation of the NL, values of parameters *r*_0_, *F*_S_ and *k*^(+)^ for HsK-CL and HsK-CL-6AA would be different from those of HsK and HsK-6AA. By adjusting *r*_0_ = 600, *F*_S_ = 5 pN, and *k*^(+)^ = 107 $s^{-1}$ (see Table 1), the calculated results for dynamics of both HsK-CL and HsK-CL-6AA such as the force dependence of velocity (Figure 6a), stepping ratio (Figure 6b), and randomness parameter (Figure 6c) are in agreement with the single-molecule data of Clancy et al. [26] and Andreasson et al. [24]. In particular, the calculated data show that about 3.3 ATP molecules consumed per forward step under no load for HsK-CL-6AA (Figure 6d), which is in quantitative agreement with the experimental data of about 3.5 [26]. The calculated data of nearly 1 ATP molecule consumed per forward step under no load for HsK-CL is also in quantitative agreement with the experimental data [27] (Figure 6d).

Taken together, *with only three adjustable parameters r*_0_, *F*_S_ and *k*^(+)^, the calculated results for both HsK-CL and HsK-CL-6AA shown in Figure 6 are in quantitative agreement with the single-molecule data. In addition, it is noted that *r*_0_ = 600 and *F*_S_ = 5 pN for HsK-CL and HsK-CL-6AA are smaller than *r*_0_ = 900 and *F*_S_ = 7 pN for HsK and HsK-6AA (see Table 1). This is understandable easily, because the mutation of the NL reduces the NL-docking energy, decreasing and increasing the probabilities for the detached ADP-head to move from the INT position to the front and rear binding sites on MT, respectively, under the backward load.

### 2.5. Dynamics of Kinesin-1 with Extended NLs at Non-Saturating ATP

At low ATP, the pathway for the chemomechanical coupling of mutant kinesin-1 with extended NLs is schematically shown in Figure 1, with *P*_0_ < 1. Since it is difficult to obtain exactly analytical solutions for the dynamics of the motor at non-saturating ATP, here we use Monte-Carlo (MC) algorithm (see Section 3) to study numerically the dynamics of the mutant kinesin-1. At non-saturating ATP, besides parameters *r*_0_, *F*_S_, *k*^(+)^, *k*^(^^−)^, and *P*_0_(0) (see above section), two additional parameters *k*_b_ and *k*_−1_ are also required to study the dynamics. Parameter *k*_b_ (also independent of force) is the second-order rate constant of ATP binding to the ϕ-head in any position on MT (whether it is in the leading or INT or trailing position) and parameter *k*_−1_ (also independent of force) is the rate constant of ATP dissociation from the leading head with the backward NL orientation (see Section 3). Note that when the head is in the trailing or INT position, without the backward NL orientation, we take zero ATP dissociation rate (see Section 3).

In our previous work [38], using MC simulations and with six parameters *r*_0_, *F*_S_, *k*^(+)^, *k*^(^^−)^, *k*_b_, and *k*_−1_, diverse and even contradictory available single-molecule data on dynamics of different species of WT kinesin-1 dimers such as squid optic lobe kinesin, DmK, and Bovine (with *P*_0_ = 1) under varying external force and ATP concentrations were reproduced well. Here, we focus on dynamics of the mutant kinesin-1 dimer with extended NLs such as HsK-CL-6AA under varying external force and ATP concentrations. For HsK-CL-6AA, we have values of *r*_0_, *F*_S_, *k*^(+)^, *k*^(^^−)^, and *P*_0_(0) (see Table 1), as given in Figure 6. To study its dynamics at non-saturating ATP, we take *k*_b_ = 3.3 ${\mu M}^{-1}s^{-1}$ and *k*_−1_ = 30 $s^{-1}$ (see Table 1).

**Figure 7:**
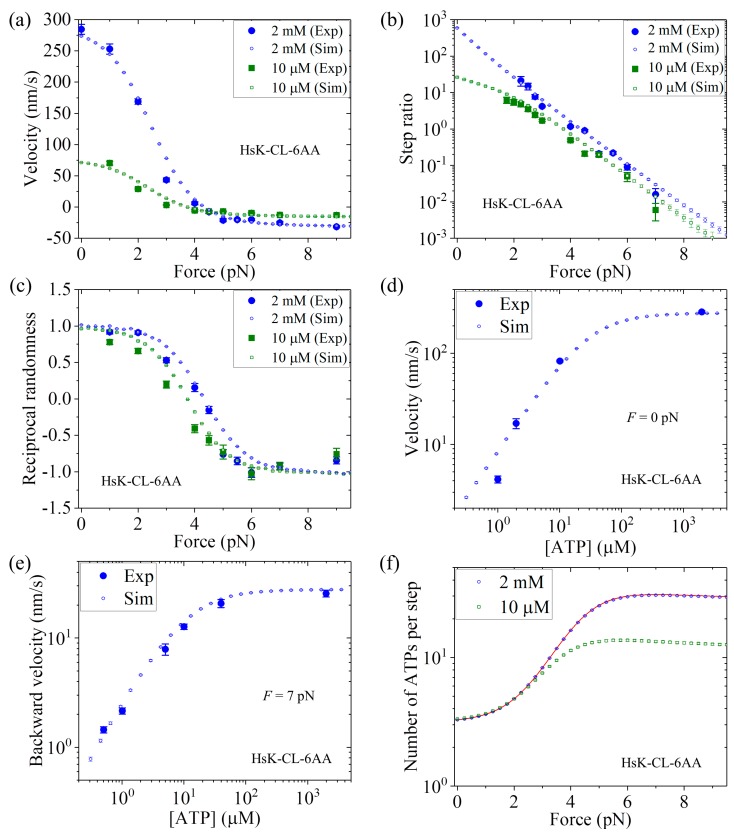
Dynamics of HsK-CL-6AA at different ATP concentrations. Unfilled symbols are MC simulated data, with error bars representing SEM (*n* = 1000), line is the analytical solution at saturating ATP, and filled symbols are experimental data taken from Clancy et al. [26] Adapted by permission from Springer Nature: Clancy, B.E.; Behnke-Parks, W.M.; Andreasson, J.O.L.; Rosenfeld, S.S.; Block, S.M. A universal pathway for kinesin stepping. *Nat. Struct. Mol. Biol.* **2011**, *18*, 1020–1027. Licence number: 4680780256064. (**a**) Velocity versus external force. (**b**) Stepping ratio versus external force. (**c**) Reciprocal randomness versus external force. (**d**) Velocity versus ATP concentration under no external force. (**e**) Backstepping velocity versus ATP concentration under backward force of 7 pN. (**f**) Number of ATP molecules consumed per step versus external force.

In Figure 7a we show the MC simulated and single-molecule results of the velocity versus *F* at 2 mM and 10 μM ATP concentrations. Figure 7b shows the MC simulated and single-molecule results of the stepping ratio versus *F* at 2 mM and 10 μM ATP concentrations using the method described in Section 3. In Figure 7c we show the MC simulated and single-molecule results of the reciprocal randomness, $R^{-1}$, versus *F* at 2 mM and 10 μM ATP concentrations. Figure 7d shows the MC simulated and single-molecule results of the velocity versus ATP concentration under no external force (i.e., *F* = 0). Figure 7e shows the MC simulated and single-molecule results of the backstepping velocity versus ATP concentration under *F* = 7 pN. Figure 7f shows the MC simulated results of the mean number of ATP molecules consumed per step (either a forward or a backward step) versus *F* at 2 mM and 10 μM ATP concentrations, where to check the MC simulation we also show the analytical solution of Equation (14) at saturating ATP (line). From Figure 7a–e, it is seen that all the simulated results are in quantitative agreement with the single-molecule data [26].

It should be mentioned here that the results for HsK-CL and HsK-CL-6AA shown in both Figure 6 and Figure 7 are calculated with the same parameters *r*_0_, *F*_S_, *k*^(+)^, *k*^(^^−)^, *k*_b_, and *k*_−1_ and an additional parameter *P*_0_(0) for HsK-CL-6AA (see Table 1). In addition, the two parameters *k*^(^^−)^ and *P*_0_(0) related to the leading head for HsK-CL and HsK-CL-6AA have the same values as those given in Figure 5 for HsK and HsK-6AA. Thus, *only five parameters* *r*_0_, *F*_S_, *k*^(+)^, *k*_b_, and *k*_−1_ are adjustable here to make all of the analytical and MC simulated results for both HsK-CL and HsK-CL-6AA shown in Figure 6 and Figure 7 be in quantitative agreement with the single-molecule data.

By contrast, using models presented in the literature to fit the single-molecule data, many more adjustable parameters are required for a species of the kinesin and moreover, different parameters values are required for the WT case and the corresponding case with extension of its NLs. For example, 10 and 13 parameters were adjustable in References [26] and [36], respectively, to fit the single-molecule data shown in Figure 7 for only HsK-CL-6AA. Moreover, with those fitted parameter values, the calculated numbers of ATP molecules consumed per forward step under no load are far away from the experimental value of 3.5 [26]. In addition, with those fitted parameters, the calculated ATPase rate of the motor during its processive movement is changed significantly as the force is varied in the small range from 0 to 4 pN (near the stall force), implying that variation of the NL strain by only 4 pN can result in a significant change in the ATPase rate. This is in contrast to the available experimental data showing that extending NLs of kinesin-1 dimer via adding different numbers (2–26) of residues in each NL (implying variation of the NL strain from about 30 pN to 0 pN [39,40]) has nearly no effect on the ATPase rate of the motor during its processive movement [27].

### 2.6. Dynamics of Kinesin-2

Up to now, we have focused on kinesin-1 homodimers. In this section, we study dynamics of kinesin-2 homodimer KIF17, comparing with the single-molecule data of Milic et al. [25]. For KIF17, the NL in each head has 17 residues. Previous all-atom MD simulations indicated that when the two heads are bound to MT the internally stretched force on the NLs has a very small value, close to zero [39,53], as in the case of HsK-CL-6AA. Thus, we also take *P*_0_ < 1 under no external force for KIF17.

**Figure 8:**
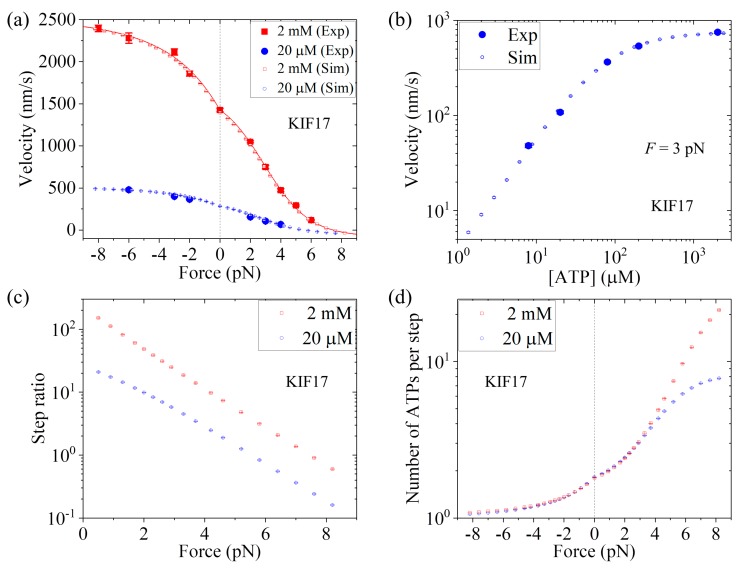
Dynamics of KIF17 at different ATP concentrations. Unfilled symbols are MC simulated data, with error bars representing SEM (*n* = 1000), line is the analytical solution at saturating ATP, and filled symbols are experimental data taken from Milic et al. [25]. (**a**) Velocity versus external force. (**b**) Velocity versus ATP concentration under backward force of 3 pN. (**c**) Stepping ratio versus external force. (**d**) Number of ATP molecules consumed per step versus external force.

First, we consider saturating ATP. We take *r*_0_ = 220, same as that for Bovine (see Table 1), and *F*_S_ = 8 pN, also close to that for Bovine (see Table 1). Then, by adjusting *k*^(+)^ = 307 $s^{-1}$, *k*^(^^−)^ = 9.8 $s^{-1}$, and *P*_0_(0) = 0.65 (see Table 1), the analytical results of the velocity versus external force (line in Figure 8a) reproduce the single-molecule data at 2 mM ATP [25] very well (Figure 8a). Then, we consider non-saturating ATP. By taking *k*_b_ = 3.3 ${\mu M}^{-1}s^{-1}$ and *k*_−1_ = 30 $s^{-1}$ (see Table 1), the MC simulated results of both the velocity versus external force *F* at [ATP] = 20 μM (Figure 8a) and the velocity versus [ATP] under *F* = 3 pN (Figure 8b) are in good agreement with the single-molecule data [25]. Moreover, in Figure 8c we show the MC simulated results of the stepping ratio versus backward force at 2 mM and 20 μM concentrations, where the stepping ratio is calculated as done in Figure 7b (see Section 3). These predicted results of Figure 8c can be test easily by using single-molecule optical trappings. In Figure 8d we show the MC simulated results of the mean number of ATP molecules consumed per step (either a forward or a backward step) versus external force at 2 mM and 20 μM ATP concentrations. From Figure 8d we see that under no external force, about 1.8 ATP molecules are consumed to make a mechanical step.

### 2.7. Power Production and Efficiency of Kinesin Dimers

One of important factors that characterize the performance of a molecular motor is the work production per unit time (power). To compare the performance among different species of kinesin motors, we calculate the power that the motor can provide at saturating ATP.

Under backward force *F* > 0, the power production can be calculated by $\dot{W}=\left\{P_{0}(0)P_{E}(F)k^{\left(+\right)}-P_{0}(F)\left[1-P_{E}(F)\right]k^{\left(-\right)}\right\}Fd$. Substitution of Equation (3) into above equation yields

(22) $$\dot{W}=\frac{P_{0}(0)r_{0}{}^{\left(1-F/F_{S}\right)}-P_{0}(F)}{r_{0}{}^{\left(1-F/F_{S}\right)}+k^{\left(+\right)}/k^{\left(-\right)}}k^{\left(+\right)}Fd$$

where *P*_0_(*F*) is calculated by Equation (8). Similarly, under forward force *F* < 0, the power production has the form

(23) $$\dot{W}=\frac{P_{0}(F)r_{0}{}^{\left(1-F/F_{S}\right)}-P_{0}(0)}{r_{0}{}^{\left(1-F/F_{S}\right)}+k^{\left(+\right)}/k^{\left(-\right)}}k^{\left(+\right)}Fd$$

where *P*_0_(*F*) is calculated by Equation (15).

**Figure 9:**
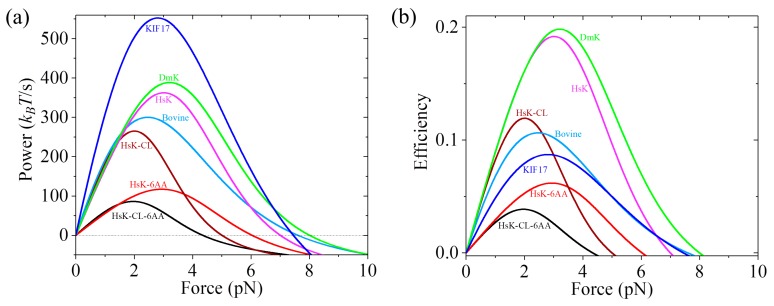
Analytical results of power production and efficiency of different kinesin motors versus backward force. (**a**) Force dependence of power production. (**b**) Force dependence of efficiency.

Using Equation (22) and with parameter values given in Table 1, the calculated results of the power versus backward force for different species of kinesin motors are shown in Figure 9a. From Figure 9a we see that the maximum power (in units of *k*_B_*T*/s) decreases in the following order: 552.6 (KIF17) > 388.5 (DmK) > 362.4 (HsK) > 300.1 (Bovine) > 265.1 (HsK-CL) > 117.0 (HsK-6AA) > 86.0 (HsK-CL-6AA). Among the seven kinesin molecular motors studied here, KIF17 has the best performance by providing the highest power of about 552.6 *k*_B_*T*/s, whereas HsK-CL-6AA has the worst performance by providing the lowest power of about 86 *k*_B_*T*/s. These results indicate that the power provided by WT kinesin-2 (KIF17) is larger than that by WT kinesin-1 (DmK, HsK, and Bovine). The power by WT kinesin-1 is larger than that by the mutant with a residue cysteine in the NL domain being mutated (HsK-CL). The extension of the NLs (HsK-6AA and HsK-CL-6AA) reduces the power relative to that without the extension of the NLs (HsK and HsK-CL).

The efficiency of kinesin motors can be calculated by $\eta =\dot{W}/\Delta \mu$, where Δμ is the energy input per unit time. In our model, $\Delta \mu =\left(k^{\left(+\right)}+k^{\left(-\right)}\right)\Delta G$, where ΔG is the free energy change provided by hydrolysis of an ATP molecule. With parameter values given in Table 1 and taking ΔG= 20 *k*_B_*T* under physiological conditions, the calculated results of η versus backward force for seven species of kinesin motors are shown in Figure 9b. It is noted that the calculated efficiency of about 16% for HsK is in agreement with the available single-molecule value determined recently at *F* = 2 pN [62]. Comparing Figure 9a with Figure 9b, we see that except for KIF17 that can provide the highest power production but has a mediate efficiency, in general, the motor that can provide a high (low) power production has a high (low) efficiency.

## 3. Methods

### 3.1. Monte Carlo Simulations

The dynamics of kinesin at non-saturating ATP is obtained using MC simulations, as used before [37,38]. We take the time step Δt = 10^−4^ s, and we have checked that reducing the time step has no effect on the simulation results. In the MC simulations, we take the following into considerations. ATP can bind to the ϕ-head in any position on MT (whether it is in the leading position or in the INT state or in the trailing position) with a constant second-order binding rate *k*_b_. When the head is in the leading position, with the backward NL orientation, ATP can dissociate with a rate constant *k*_−1_. When the head is in the trailing position or in the INT state, without the backward NL orientation, ATP can dissociate with a rate constant *k*_−2_ (for simplicity, we take *k*_−2_ = 0 throughout, implying that the rate constant of ATP dissociation from the head without the backward NL orientation is very low). This position-dependent ATP-binding efficiency (with *k*_−1_ >> *k*_−2_ = 0) is consistent with the single-molecule data of Dogan et al. [63] showing a lower efficiency of ATP binding to *E. coli* kinesin head with the backward NL orientation than that without the backward NL orientation. Rate constants *k*_b_ and *k*_−1_ are independent of the force *F* and NL length. In the trailing position the rate constant of ATPase activity (i.e., ATP transition to ADP) of the head is *k*^(+)^, independent of the force *F* and NL length, while in the leading position is *k*^(^^−)^, also independent of the force *F* and NL length. When an ATPase activity occurs in the trailing head the ADP-head either still binds to the rear binding site on MT with probability 1–*P*_0_ or moves immediately forward to the INT position with probability *P*_0_. When an ATPase activity occurs in the leading head the ADP-head either still binds to the front binding site on MT with probability 1–*P*_0_ or moves immediately backward to the INT position with probability *P*_0_. The force dependence of probability *P*_0_(*F*) is calculated by Equations (8) and (15). If the ADP-head still binds to MT, ADP is released immediately, which is followed by ATP binding. In the INT state, when the MT-bound head is in ATP state its NL docks immediately, and then the tethered ADP-head either moves immediately forward to the front binding site on MT with probability *P*_E_ or moves immediately backward to the rear binding site on MT with probability 1–*P*_E_. The dependence of probability *P*_E_ on *F* is calculated by Equation (3). Once the ADP-head binding to MT, ADP is released immediately. Here, we perform MC simulations for HsK-CL-6AA and for KIF17 at non-saturating ATP, with the parameter values: *r*_0_ = 600, *F*_S_ = 5 pN, *k*^(+)^ = 107 $s^{-1}$, *k*^(^^−^^)^ = 4 $s^{-1}$, *P*_0_(0) = 0.33, *k*_b_ = 3.3 ${\mu M}^{-1}s^{-1}$, and *k*_−__1_ = 30 $s^{-1}$ for HsK-CL-6AA, and *r*_0_ = 220, *F*_S_ = 8 pN, *k*^(+)^ = 307 $s^{-1}$, *k*^(^^−^^)^ = 9.8 $s^{-1}$, *P*_0_(0) = 0.65, *k*_b_ = 3.3 ${\mu M}^{-1}s^{-1}$, and *k*_−__1_ = 30 $s^{-1}$ for KIF17 (see Table 1). The program for the MC simulations can be downloaded from github (https://github.com/think92/kinetic-model-for-kinesin).

### 3.2. Methods for Calculation of Stepping Ratio

At non-saturating ATP, the stepping ratio is calculated as follows. As mentioned above, the interaction between the bead attached to the coiled-coil stalk and kinesin dimer acts only on the NL of the head that has a larger distance to the bead. In addition, in the single-molecule optical trapping assays the position of the motor is dictated by that of the bead. Thus, under the backward force on the bead, when one kinesin head binds to MT and the other head moves between the rear binding site on MT and the INT position no movement of the bead can be detected, when the other head moves from the INT position to the front binding site on MT a forward step of the bead can be detected, and when the other head moves from the front binding site on MT to the INT position a backward step of the bead can be detected. Besides, to directly compare with the single-molecule experimental data that were recorded at 2 kHz [26], when the other head moves from one to another position, which can be detected by the movement of the bead, only the head can stay at the latter position for a time period longer than 0.5 ms can the motor be considered to make a step.

At saturating ATP, since the detached head cannot stay at the INT position for a time period longer than 0.5 ms, the stepping ratio calculated with the method described above should be the same as that calculated based on the change of the center-of-mass position of the dimer (see Section S3 in Supplementary Information for detailed discussion). Note that the analytical results shown in Figure 3a,b, Figure 5b and Figure 6b were obtained based on the change of the center-of-mass position of the dimer.

## 4. Concluding Remarks

A general kinetic model is presented for the chemomechanical coupling of kinesin dimers without and with extension of the NLs. Compared to the previous models, the novelty of the current model is that the ATPase activity of the kinesin head is independent of both the external force and the internal tension on the NLs or length of the NLs, implying that for the same species of kinesin both the WT case and the mutant case with extension of its NLs have the same rate constants of the ATPase activity. Based on the model, an analytical theory is presented for the dynamics of various kinesin dimers, such as WT kinesin-1, kinesin-1 with mutated residue in the NL domain, kinesin-1 with extension of the NLs, and WT kinesin-2, under varying force at saturating ATP. At non-saturating ATP the dynamics is studied numerically using the MC algorithm. With only a few adjustable parameters, diverse available single-molecule data can be explained quantitatively and consistently. By contrast, using the previous models, many more adjustable parameters are required. Although here we have made studies for only kinesin-1 and kinesin-2, our analytical theory can also be applicable to other families of processive kinesin dimers. For example, using Equation (5), the available single-molecule data on the force dependence of velocity for kinesin-5 dimer and kinesin-3 dimer at saturating ATP can also be explained well (see Figure S2 in Supplementary Information).

As mentioned in Section 2.1, our model is built up on the basis of the available structural, experimental and computational evidence. Moreover, based on the model and with only a few adjustable parameters, diverse available experimental data on dynamics of various kinesin homodimers with and without extension of the NLs can be explained quantitatively and consistently. All these give support to the model.

In order to further verify the model, it is hoped that the predicted results can be tested in future experiments. For example, using single-molecule optical trapping techniques, the predicted results on the force dependence of stepping ratio for HsK and HsK-6AA (Figure 5b), HsK (Figure 6b) and KIF17 (Figure 8c) can be tested. Using single-molecule optical trapping techniques complemented with using a fluorescent ATP analogue (deac-aminoATP), we can simultaneously monitor processive stepping and nucleotide binding/dissociation under different forces, similar to that used by Sakamoto et al. [64] to study the chemomechanical coupling of myosin-V. Thus, the predicted results on the force dependence of the number of ATPs consumed per step, which is equivalent to the inverse of the chemomechanical coupling ratio, for different species of kinesin motors (Figure 6d, Figure 7f and Figure 8d) can be tested. The straightforward comparison of the predicted results with the experimental data is critical to prove the model.

## Figures and Tables

**Table 1:** Values of parameters for different species of kinesin motors.

Parameters	DmK	Bovine	HsK	HsK-6AA	HsK-CL	HsK-CL-6AA	KIF17
*r*_0_	890 ± 120	220 ± 68	900	900	600 ± 50	600 ± 50	220
*F*_S_ (pN)	8 ± 0.3	7.6 ± 1.1	7	7	5 ± 0.4	5 ± 0.4	8
*k*^(+)^ ($s^{-1}$)	95 ± 1	138 ± 20	90.5 ± 7.1	90.5 ± 7.1	107 ± 3	107 ± 3	307 ± 22
*k*^(^^−)^ ($s^{-1}$)	3 ± 0.4	3 ± 1.1	4 ± 0.5	4 ± 0.5	4	4	9.8 ± 1.6
*P*_0_(0)	-	-	-	0.33 ± 0.02	-	0.33	0.65 ± 0.1
*k*_b_ (${\mu M}^{-1}s^{-1}$)	-	-	-	-	-	3.3	3.3
*k*_−__1_ ($s^{-1}$)	-	-	-	-	-	30	30

Symbol “-” denotes that the value is not required in the calculation in the main text. Errors are estimated from least-squares fittings to the available experimental data at saturating ATP.

## Supplementary Materials

Supplementary materials can be found at https://www.mdpi.com/1422-0067/20/19/4911/s1.
