# Supplementary material for: A Generalized Kinetic Model for Coupling between Stepping and ATP Hydrolysis of Kinesin Molecular Motors
Source: Int J Mol Sci. 2019 Oct 3;20(19):4911. doi: 10.3390/ijms20194911 (PMC6801755; doi:10.3390/ijms20194911)
Supplement: Supplementary file 1 [file ijms-20-04911-s001.pdf]

Supplementary Information

A Generalized Kinetic Model for Coupling between Stepping and ATP Hydrolysis of Kinesin Molecular Motors

Ping Xie [1,2,*], Si-Kao Guo [2], Hong Chen [1]

1. School of Materials Science and Energy Engineering, FoShan University, Guangdong, 528000, China

2. Institute of Physics, Chinese Academy of Sciences, Beijing 100190, China

*Corresponding author:

E-mail address: pxie@aphy.iphy.ac.cn

Supplementary Text

S1. Derivation of equation for the force dependence of stepping ratio for WT kinesin at saturating ATP

First, we consider the simple case of NL-docking energy E_D = 0. Based on our model (Figure 2), under F = 0 the movement of the detached ADP-head from the INT position to the front binding site on MT requires overcoming the same energy barrier E_0 as that to the rear binding site, where E_0 results from the weak binding energy, E_I, between the two heads and the energy change, ΔE_{NL}, arising from the stretching of the NLs. Considering $d^{(+)} \approx d^{(-)}$, the rates for the detached ADP-head to move to the front binding site and to the rear binding site can be approximately calculated by $k_{\text{F}} = k_{\text{R}} = C\exp\left(-\beta E_0\right)$, where C is a constant. The probability P_E can then be calculated by

$$P_{\text{E}} = \frac{k_{\text{F}}}{k_{\text{F}} + k_{\text{R}}}. \tag{S1}$$

Then, consider a non-zero NL-docking energy E_D. Under F = 0, the movement of the detached ADP-head from the INT position to the front binding site on MT still requires overcoming the energy barrier $E_0^{(\text{F})} = E_0$, while the movement to the rear

binding site on MT requires overcoming the energy barrier $E_0^{(\mathrm{R})}$, which can be approximately calculated by $E_0^{(\mathrm{R})} = E_0 + E_\mathrm{D}$. Approximately, the rate for the detached ADP-head to move to the front binding site on MT can still be calculated by $k_\mathrm{F} = C\exp\left(-\beta E_0^{(\mathrm{F})}\right)$ and the rate for the detached ADP-head to move to the rear binding site on MT can be calculated by $k_\mathrm{R} = C\exp\left(-\beta E_0^{(\mathrm{R})}\right)$. Under $F > 0$, since F acts only the head in the leading position, we approximately have $k_\mathrm{F} = C\exp\left[-\beta\left(E_0^{(\mathrm{F})} + Fd^{(+)}\right)\right]$ and $k_\mathrm{R} = C\exp\left(-\beta E_0^{(\mathrm{R})}\right)$, which can be rewritten as

$$k_\mathrm{F} = C_1 \exp\left(-\beta F d^{(+)}\right), \tag{S2}$$

$$k_\mathrm{R} = C_1 \exp\left(-\beta E_\mathrm{D}\right), \tag{S3}$$

where $C_1 = C\exp\left(-\beta E_0\right)$ is independent of F. Substituting Eqs. (S2) and (S3) into Eq. (S1), we have

$$P_\mathrm{E} = \frac{\exp\left[\beta\left(E_\mathrm{D} - Fd^{(+)}\right)\right]}{\exp\left[\beta\left(E_\mathrm{D} - Fd^{(+)}\right)\right] + 1}. \tag{S4}$$

From Figure 2g with $P_0 = 1$ the stepping ratio can be calculated by $r = P_\mathrm{E} k^{(+)} \big/ \left[\left(1 - P_\mathrm{E}\right) k^{(-)}\right]$, with which and Eqs. (S2) and (S3) we have following force dependence of stepping ratio, $r = \left(k^{(+)}/k^{(-)}\right)\exp\left[\beta\left(E_\mathrm{D} - Fd^{(+)}\right)\right]$. The equation can be rewritten as

$$r = r_0 \exp\left(-\beta F d^{(+)}\right), \tag{S5}$$

where $r_0 = \left(k^{(+)}/k^{(-)}\right)\exp\left(\beta E_\mathrm{D}\right)$ is the stepping ratio under $F = 0$. Similarly, under $F < 0$ we have

$$r = r_0 \exp\left(-\beta F d^{(-)}\right). \tag{S6}$$

where $r_0 = \left(k^{(+)}/k^{(-)}\right)\exp\left(\beta E_\mathrm{D}\right)$, as in Eq. (S5). Taking $\alpha^{(+)} = \beta d^{(+)}$ and $\alpha^{(-)} = \beta d^{(-)}$ and with $\alpha^{(+)} \approx \alpha^{(-)} = \alpha$, Eqs. (S5) and (S6) can be rewritten as

$$r = r_0^{\left(1 - F/F_\mathrm{S}\right)}, \tag{S7}$$

$$r_0 = \frac{k^{(+)}}{k^{(-)}} \exp\left(\beta E_\mathrm{D}\right), \tag{S8}$$

where $F_\mathrm{S} = \log\left(r_0\right)/\alpha$.

S2. Equations for dependence of WT kinesin stepping dynamics upon backward force and temperature at saturating ATP

In this section, we consider only the backward force ($F > 0$) acting on WT kinesin dimer and saturating ATP. The pathway is shown in Figure 2, with $P_0 = 1$.

The dependence of the ATPase rate on temperature T can be calculated by using transition state theory formula

$$k^{(+)} = k_0 \exp\left(-\frac{E_a^{(+)}}{k_B T} \right), \tag{S9}$$

$$k^{(-)} = k_0 \exp\left(-\frac{E_a^{(-)}}{k_B T} \right), \tag{S10}$$

where $k_0 \approx 6 \times 10^{12}\ s^{-1}$ is a universal constant that is equivalent to the ATPase rate with zero activation energy [S1–S3], $E_a^{(+)}$ is the activation energy of ATPase activity in the trailing kinesin head and $E_a^{(-)}$ is the activation energy of ATPase activity in the leading kinesin head. From Eq. (S4) under $F > 0$, probability P_E can be calculated by

$$P_E = \frac{\exp\left(\frac{E_D}{k_B T} \right) \exp\left(-\frac{F d^{(+)}}{k_B T} \right)}{\exp\left(\frac{E_D}{k_B T} \right) \exp\left(-\frac{F d^{(+)}}{k_B T} \right) + 1}. \tag{S11}$$

From Figure 2g with $P_0 = 1$, the forward and backward stepping rates have forms, $\omega_f = k^{(+)} P_E$ and $\omega_b = k^{(-)} (1 - P_E)$. Substituting Eqs. (S9) – (S11) into these two equations, we have the following force and temperature dependence of forward and backward stepping rates

$$\omega_f = k_0 \frac{\exp\left(-\frac{E_a^{(+)}}{k_B T} \right)}{\exp\left(-\frac{F d^{(+)}}{k_B T} \right) + \exp\left(-\frac{E_D}{k_B T} \right)} \exp\left(-\frac{F d^{(+)}}{k_B T} \right), \tag{S12}$$

$$\omega_b = k_0 \frac{\exp\left(-\frac{E_a^{(-)} + E_D}{k_B T} \right)}{\exp\left(-\frac{F d^{(+)}}{k_B T} \right) + \exp\left(-\frac{E_D}{k_B T} \right)}. \tag{S13}$$

The stepping ratio can be calculated by $r = \omega_f / \omega_b$, which can be rewritten as

$$r = r_0 \exp\left(-\frac{Fd^{(+)}}{k_B T}\right), \tag{S14}$$

where $r_0 = \exp\left[\left(E_D + E_a^{(-)} - E_a^{(+)}\right)/\left(k_B T\right)\right]$ is the stepping ratio under no force, with $\ln\left(r_0\right)$ being inversely proportional to *T*. Eq. (S14) can be rewritten as

$$r = r_0^{(1-F/F_S)}, \tag{S15}$$

where the stall force $F_S = k_B T \ln\left(r_0\right)/d^{(+)} = \left(E_D + E_a^{(-)} - E_a^{(+)}\right)/d^{(+)}$ is independent of *T*, in good agreement with the available single-molecule data of Kawaguchi and Ishiwata [S4].

The velocity of the motor can be calculated by

$$v = \left(\omega_f - \omega_b\right) d . \tag{S16}$$

As derived in the main text, the mean dwell time between two mechanical steps can be calculated by

$$T_d = \frac{1}{k^{(+)} P_E + k^{(-)}(1 - P_E)} . \tag{S17}$$

As $\omega_f = k^{(+)} P_E$ and $\omega_b = k^{(-)}\left(1 - P_E\right)$, Eq. (S17) can be rewritten as

$$T_d = \frac{1}{\omega_f + \omega_b} . \tag{S18}$$

By adjusting $E_a^{(+)}$ = 94.5 pN nm, $E_a^{(-)}$ = 119 pN nm, E_D = 0.4 pN nm and $d^{(+)}$ = 2.65 nm (see Table S1), with Eqs. (S12), (S13) and (S18) the single-molecule data of Taniguchi et al. [S5] on the dependence of the dynamics of bovine brain kinesin upon backward force *F* and temperature *T* can be reproduced quantitatively (Figure 4). In other words, with only four adjustable parameters, all the available single-molecule data of Taniguchi et al. [S5] on the dependence of forward and backward stepping dynamics upon backward force and temperature can be fitted well.

The fitted value of the activation energy of ATPase activity, $E_a^{(+)}$ = 94.5 pN nm ≈ 14 kcal/mol, is consistent with the value of about 94.64 pN nm [S3] or 14.4 kcal/mol [S6] or 14.6 kcal/mol [S2] determined in the literature. It is also in the range of about 47.8 kJ/mol (11.4 kcal/mol), 50 kJ/mol (12 kcal/mol) and 65 kJ/mol (15.6 kcal/mol) determined by Adio and Woehlke [S7], Kawaguchi and Ishiwata [S4,S8] and Hong et al. [S9], respectively, from their experimental data on the temperature

dependence of kinesin velocity. As defined in the main text, $k^{(+)}$ is the rate of ATP hydrolysis and Pi release in the trailing head at saturating ATP (see Figure 2a). As Pi release is rate limiting in the two chemical reaction steps, from Eq. (S9) it is expected that the high activation energy $E_a^{(+)}$ could be attributed mainly to the large conformational change of the trailing head associated with Pi release. The fitted value of $E_a^{(+)}$ for the trailing head that is smaller than that of $E_a^{(-)}$ for the leading head (see Table S1) can be understood as follows. As mentioned in the main text, for the trailing head the NL has the forward orientation, which can interact with the head, and by contrast, for the leading head the NL has the backward orientation, which has no interaction with the head. With the interaction with the NL the trailing head could have a smaller Pi-release associated conformational change than the leading head without the interaction with the NL, resulting in $E_a^{(+)}$ to be lower than $E_a^{(-)}$.

The fitted value of $d^{(+)}$ = 2.65 nm (see Table S1) is close to $d^{(+)}$ = 2.9 nm fitted to the curve in Figure 3b. From the fitted values of $E_a^{(+)}$, $E_a^{(-)}$ and E_D, we have the stepping ratio under no force, r_0 = $\exp\left[\left(E_D + E_a^{(-)} - E_a^{(+)}\right)/\left(k_B T\right)\right]$ = 434 at room temperature T = 298 K, which is close to that fitted to the single-molecule data of Nishiyama et al. [S9] (Figure 3b). In particular, the fitted value of E_D = 0.4 pN nm implies that the free-energy change of NL docking is very small, consistent with the available experimental result [S11].

Table S1. Parameter values used for fitting experimental data of Taniguchi et al. [S5] for bovine brain kinesin

$E_a^{(+)}$ (pN nm)	$E_a^{(-)}$ (pN nm)	E_D (pN nm)	$d^{(+)}$ (nm)
94.5	119	0.4	2.65

S3. Comparison of stepping ratios calculated with different methods

Here, we consider two methods to calculate the stepping ratio under the backward force. One method (called Method I) is based on the change of the position in the

center of mass (COM) of the dimer, as done in the derivation of Eqs. (1) and (9) in the main text. When the COM of the dimer was moved forward by d = 8.2 nm it is considered that a forward step was made, while when the COM of the dimer was moved backward by d = 8.2 nm it is considered that a backward step was made. In this method the real numbers of forward and backward steps of the motor are counted. Another method (called Method II) is based on the one used in the single-molecule optical trapping experiments, as described in Methods section in the main text. Method II is briefly re-described as follows. Under the backward force on the bead, when one kinesin head binds to MT and the other head moves between the rear binding site on MT and the INT position no movement of the bead can be detected, when the other head moves from the INT position to the front binding site on MT a forward step of the bead can be detected, and when the other head moves from the front binding site on MT to the INT position a backward step of the bead can be detected. Besides, since in the single-molecule experiments the data were recorded at 2 kHz [S12], when the other head moves from one to another position, which can be detected by the movement of the bead, only the head can stay at the latter position for a time period longer than 0.5 ms can the motor be considered to make a step.

At saturating ATP, the stepping ratio versus backward force for HsK-CL-6AA can be calculated using Eq. (9) for Method I. With parameters given in Table 1 for HsK-CL-6AA, the calculated results of the stepping ratio versus backward force using Eq. (9) are shown in Figure S3a (line). For Method II we use MC algorithm (see main text). The MC simulated results of the stepping ratio versus backward force at 2 mM ATP for HsK-CL-6AA are also shown in Figure S3a (dots). It is seen that the results for the two methods are nearly identical. This can be explained as follows. Since at saturating ATP (2 mM) the detached head cannot stay at the INT position for a time period longer than 0.5 ms, for both methods a forward step corresponds to a head moving from the rear to front binding site on MT while a backward step corresponds to a head moving from the front to rear binding site. At low ATP, we use MC algorithm to simulate the stepping ratio for both Method I and Method II. The MC simulated results at 10 μM ATP for HsK-CL-6AA are shown in Figure S3b. It is seen that the two methods give very different results under low backward forces. This can be explained as follows. At low ATP, the detached head can stay at the INT position for a time period almost always longer than 0.5 ms. Thus, for Method II,

under the backward force a forward step corresponds to the detached head moving from the INT position to the front binding site on MT while a backward step corresponds to the detached head moving from the front binding site on MT to the INT position. By contrast, for Method I, a forward step still corresponds to a head moving from the rear to front binding site on MT while a backward step still corresponds to a head moving from the front to rear binding site.

In Figure S3, we studied the kinesin dimer with $P_0 < 1$ when $F = 0$. Then, we study the kinesin dimer with $P_0 = 1$. For example, we consider Bovine. As shown in Table 1, we have $r_0 = 220$, $F_S = 7.6$ pN, $k^{(+)} = 138\ \mathrm{s}^{-1}$ and $k^{(-)} = 3\ \mathrm{s}^{-1}$ for the case at saturating ATP. In addition, we take $k_b = 6\ \mu\mathrm{M}^{-1}\mathrm{s}^{-1}$ and $k_{-1} = 180\ \mathrm{s}^{-1}$ for the case at low ATP. In Figure S4a we show the MC simulated and single-molecule results of the velocity versus F at 1 mM and 10 μM ATP concentrations. In Figure S4b we show the MC simulated results of stepping ratio versus F at 1 mM and 10 μM ATP concentrations for Method I and compare with the single-molecule results. In Figure S4c we show the MC simulated results of stepping ratio versus F at 1 mM and 10 μM ATP concentrations for Method II and compare with the single-molecule results. From Figure S4a – c, it is seen that all the simulated results are in agreement with the experimental results. Nevertheless, the two methods give different stepping ratios at low ATP and under low backward forces (Figure S4d).

References

[S1] Glennon T.M., Villa J., Warshel A. (2000) How does GAP catalyze the GTPase reaction of Ras?: A computer simulation study. Biochemistry 39, 9641–9651.

[S2] Grigorenko B.L., Rogov A.V., Topol I.A., Burt S.K., Martinez H.M., Nemukhin A.V. (2007) Mechanism of the myosin catalyzed hydrolysis of ATP as rationalized by molecular modeling. Proc. Natl Acad. Sci. U. S. A. 104, 7057–7061.

[S3] Xie P. (2019) Modeling DNA unwinding by AddAB helicase-nuclease and modulation by Chi sequences: comparison with AdnAB and RecBCD. Cell Mol. Bioeng. 12, 179–191.

[S4] Kawaguchi K., Ishiwata S. (2000) Temperature dependence of force, velocity, and processivity of single kinesin molecules. Biochem. Biophys. Res. Commun. 272, 895–899.

[S5] Taniguchi Y., Nishiyama M., Ishii Y., Yanagida T. (2005) Entropy rectifies the Brownian steps of kinesin. Nat. Chem. Biol. 1, 342–347.

[S6] Bedale W.A, Cox M.M. (1996) Evidence for the coupling of ATP hydrolysis to the final (extension) phase of RecA protein-mediated DNA strand exchange. J. Biol. Chem. 271, 5725–5732.

[S7] Adio S., Woehlke G. (2009) Properties of the Kinesin-3 NcKin3 motor domain and implications for neck function. FEBS Journal 276, 3641–3655.

[S8] Kawaguchi K., Ishiwata S. (2001) Thermal Activation of Single Kinesin Molecules With Temperature Pulse Microscopy. Cell Motility and the Cytoskeleton 49, 41–47.

[S9] Hong W., Takshak A., Osunbayo O., Kunwar A., Vershinin M. (2016) The Effect of Temperature on Microtubule-Based Transport by Cytoplasmic Dynein and Kinesin-1 Motors. Biophysical Journal 111, 1287–1294.

[S10] Nishiyama M., Higuchi H., Yanagida T. (2002) Chemomechanical coupling of the forward and backward steps of single kinesin molecules. Nat. Cell Biol. 4, 790–797.

[S11] Rice S., Cui Y, Sindelar C., Naber N., Matuska M., Vale R., Cooke R. (2003) Thermodynamic properties of the kinesin neck region docking to the catalytic core. Biophys J. 84, 1844–1854.

[S12] Clancy B.E., Behnke-Parks W.M., Andreasson J.O.L., Rosenfeld S.S., Block S.M. (2011) A universal pathway for kinesin stepping. Nat. Struct. Mol. Biol. 18, 1020–1027.

[S13] Valentine M.T., Fordyce P.M., Krzysiak T.C., Gilbert S.P., Block S.M. (2006). Individual dimers of the mitotic kinesin motor Eg5 step processively and support substantial loads in vitro. Nat. Cell Biol. 8, 470-476.

[S14] Tomishige M., Klopfenstein D.R., Vale R.D. (2002) Conversion of Unc104/KIF1A kinesin into a processive motor after dimerization. Science 297, 2263-2267.

Supplementary Figures

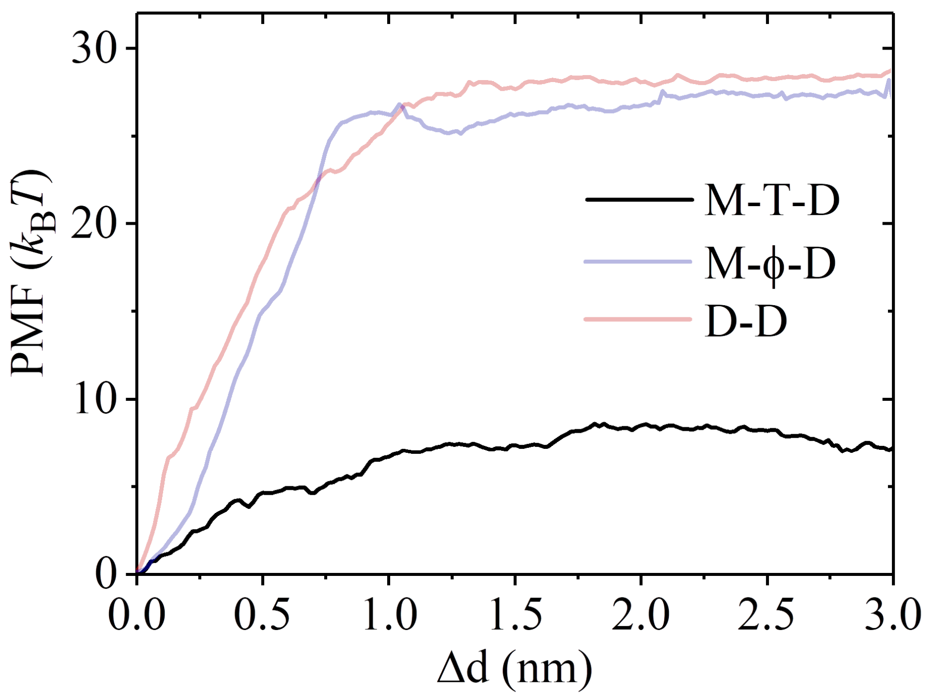

Figure S1. All-atom MD simulation results of the binding energy (characterized by potential of mean force, PMF) between the MT-bound head and detached ADP-head versus the change in the distance (Δd) between the two heads. The plot is reproduced from manuscript (Shi, X-X., Guo S.-K., Wang P.-Y., Chen H, Xie P., to be published in Proteins). M-T-D represents that the MT-bound head is in ATP state with docked NL and the detached head is in ADP state, M-ϕ-D represents that the MT-bound head is in nucleotide-free (ϕ) state with undocked NL and the detached head is in ADP state, and D-D represents that the two heads are in ADP state with undocked NLs. PMF was calculated using standard MD simulations, as well as center-of-mass pulling and umbrella sampling simulations. The results indicate that the MT-bound ATP-head with docked NL has a much weaker binding energy with the detached ADP-head than the MT-bound ϕ- or ADP-head with undocked NL.

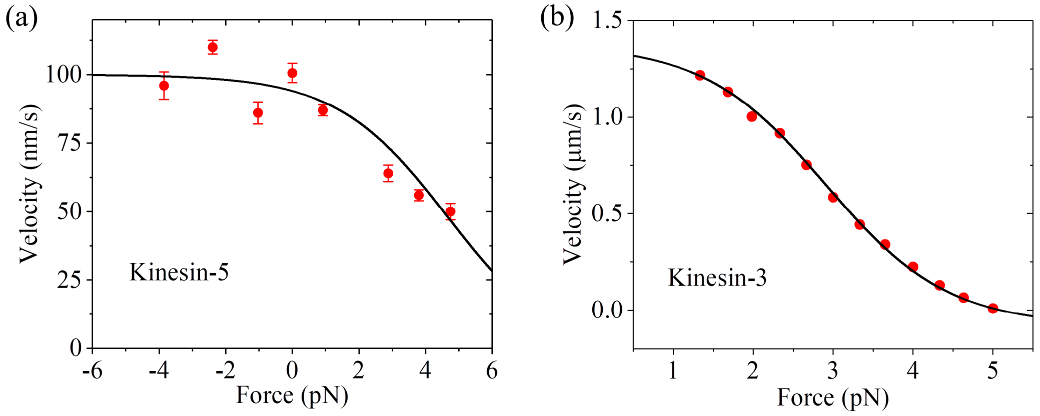

Figure S2. Dynamics of kinesin-5 dimer and kinesin-3 dimer at saturating ATP. (a) Force dependence of velocity for kinesin-5 dimer. Symbols represent experimental data at 2 mM ATP taken from Valentine et al. [S13]. Line represents analytical solution of Eq. (5), with $r_0 = 400$, $F_S = 10$ pN, $k^{(+)} = 12.2\ \ s^{-1}$ and $k^{(-)} = 0.5\ \ s^{-1}$. **(b)** Force dependence of velocity for kinesin-3 dimer. Symbols represent experimental data for dimerized U356-Kstalk-GFP taken from Tomishige et al. [S14]. Line represents analytical solution of Eq. (5), with $r_0 = 800$, $F_S = 5.1$ pN, $k^{(+)} = 168\ \ s^{-1}$ and $k^{(-)} = 9.5\ \ s^{-1}$.

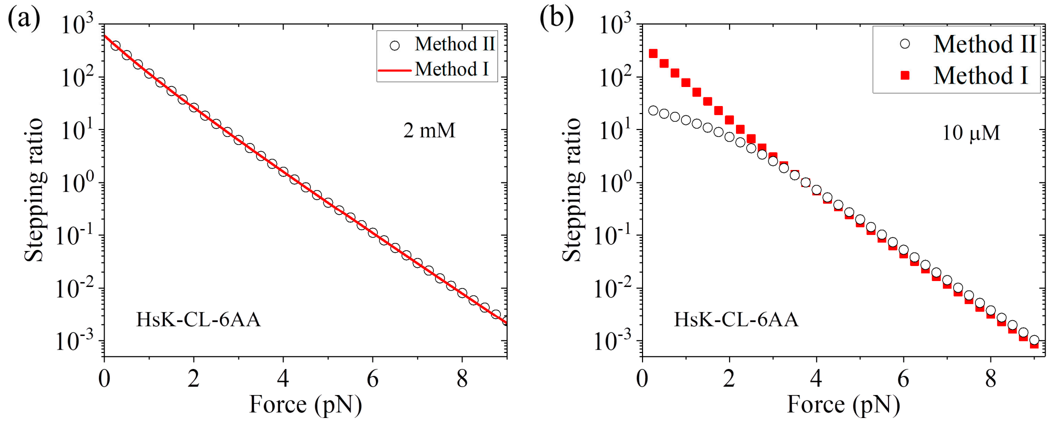

Figure S3. Comparison of stepping ratios for HsK-CL-6AA calculated with different methods. (a) Results of the stepping ratio versus backward force at saturating ATP (2 mM). Line is the analytical solution of Eq. (9) for Method I, while unfilled circles are MC simulated results at 2 mM for Method II. **(b)** Results of the stepping ratio versus backward force at 10 μM ATP. Filled squares are MC simulated results for Method I, while unfilled circles are MC simulated results for Method II.

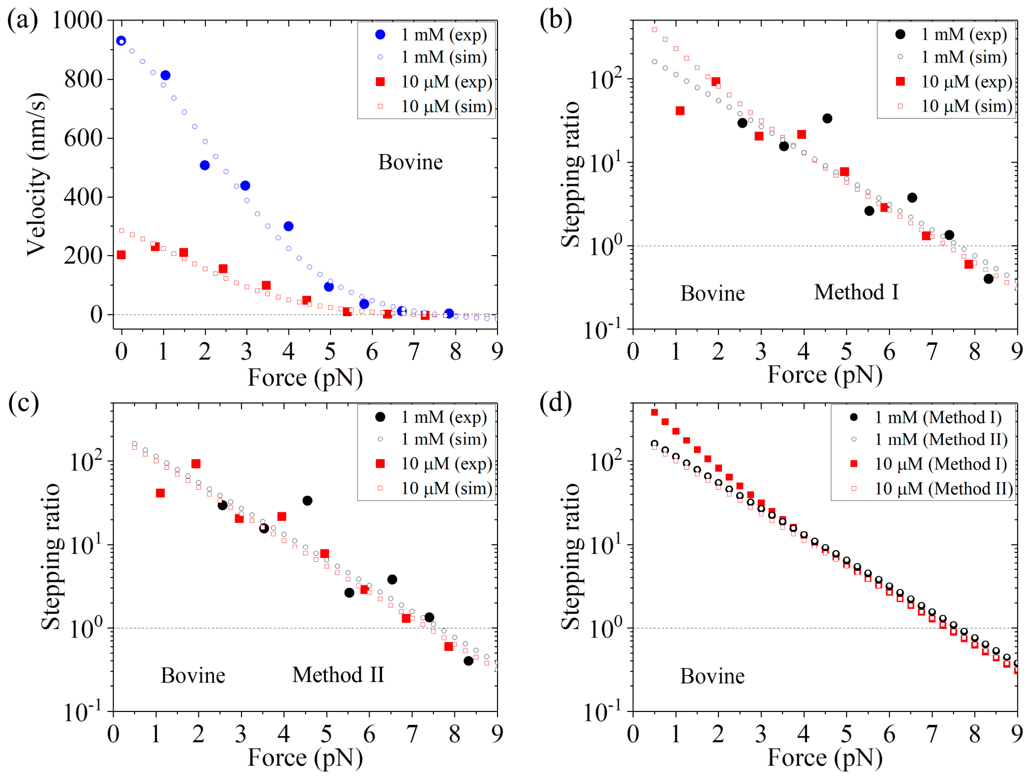

Figure S4. Comparison of stepping ratios for Bovine calculated with different methods. Unfilled symbols are MC simulated results, while filled symbols are single-molecule data taken from Nishiyama et al. [S10]. **(a)** Velocity versus backward force at 1 mM and 10 μM ATP concentrations. **(b)** Stepping ratio versus backward force at 1 mM and 10 μM ATP concentrations for Method I. **(c)** Stepping ratio versus backward force at 1 mM and 10 μM ATP concentrations for Method II. **(d)** MC simulated results of stepping ratio versus backward force at 1 mM and 10 μM ATP concentrations for Method I and Method II.
